# The Stroop legacy: A cautionary tale on methodological issues and a proposed spatial solution

**DOI:** 10.3758/s13428-023-02215-0

**Published:** 2023-08-24

**Authors:** Giada Viviani, Antonino Visalli, Maria Montefinese, Antonino Vallesi, Ettore Ambrosini

**Affiliations:** 1 Department of Neuroscience, University of Padova, 35121 Padova, Italy; 2 Padova Neuroscience Center, University of Padova, Padova, Italy; 3 IRCCS San Camillo Hospital, Venice, Italy; 4 Department of Developmental and Social Psychology, University of Padova, Padova, Italy; 5 Department of General Psychology, University of Padova, Padova, Italy

**Keywords:** Stroop task, Spatial stroop, Dimensional overlap, Multiple loci account, Cognitive control

## Abstract

The Stroop task is a seminal paradigm in experimental psychology, so much that various variants of the classical color–word version have been proposed. Here we offer a methodological review of them to emphasize the importance of designing methodologically rigorous Stroop tasks. This is not an end by itself, but it is fundamental to achieve adequate measurement validity, which is currently hindered by methodological heterogeneity and limitations. Among the several Stroop task variants in the literature, our methodological overview shows that the spatial Stroop task is not only a potentially methodologically adequate variant, which can thus assure measuring the Stroop effect with the required validity, but it might even allow researchers to overcome some of the methodological limitations of the classical paradigm due to its use of verbal stimuli. We thus focused on the spatial Stroop tasks in the literature to verify whether they really exploit such inherent potentiality. However, we show that this was generally not the case because only a few of them (1) are purely spatial, (2) ensure both all the three types of conflicts/facilitations (at the stimulus, response, and task levels) and the dimensional overlaps considered fundamental for yielding a complete Stroop effect according to the multiple loci account and Kornblum’s theory, respectively, and (3) controlled for low-level binding and priming effects that could bias the estimated Stroop effect. Based on these methodological considerations, we present some examples of spatial Stroop tasks that, in our view, satisfy such requirements and, thus, ensure producing complete Stroop effects.

## Introduction

One of the most influential and widely used experimental paradigms in cognitive psychology is the Stroop task (Stroop, 1935). In its most common version, known as the color–word Stroop task, participants are presented with words that denote a color printed in either the same or a different ink color and are required to identify the ink color of the presented word regardless of its meaning. Crucially, the ink color in which the word is displayed and the meaning of the same word can either match (congruent trials, e.g., GREEN displayed in green ink: GREEN_green_) or not (incongruent trials, e.g., GREEN displayed in blue ink: GREEN_blue_). The critical measure is the so-called Stroop effect (SE), which refers to the robust performance decline in incongruent as compared to congruent trials, which is commonly attributed to the interference between reading and color naming (e.g., MacLeod, 1991; Stroop, 1935). Despite being the most classic Stroop task account, this explanation is incomplete and insufficient, as will be discussed profusely throughout this work. Indeed, the need to conduct this review arises precisely from the widespread belief that, in order to obtain a Stroop effect, the only basic requirement is to administer a task with congruent and incongruent stimuli, which is however incorrect or, at least, not sufficient.

Before delving into theoretical and methodological issues, we first provide the reader with definitions of some basic concepts, starting with the Stroop effect, which is commonly computed as the difference in the response time (RT) between incongruent (I) and congruent (C) trials (formally, SE = RT[I] – RT[C]). When congruent trials are used as the baseline condition against which to compare RT on incongruent trials, the SE has also been referred to as the Stroop congruency effect (e.g., Egner et al., 2010), Stroop interference effect (e.g., Leung et al., 2000), or total Strop effect (e.g., Brown et al., 1998). Alternatively, neutral trials (e.g., a color-neutral word or a non-word letter string displayed in green ink: CAT_green_ or XXX_green_, respectively), can be used as the baseline condition, allowing one to partition the SE into Stroop interference (SI) and facilitation (SF) effects. The former, calculated as the difference in RT between incongruent and neutral trials (N) (formally, SI = RT[I] – RT[N]), refers to a worse performance for incongruent (I) than neutral words (N); the latter, computed as the difference in RT between neutral and congruent trials (formally, SF = RT[N] – RT[C]), refers to a better performance for congruent than neutral words. The algebraic sum of SI and SF corresponds to the full SE (formally, SE = SI + SF).

To successfully complete the Stroop task, some form of cognitive control, namely the ability to regulate thoughts and actions in accordance with internally maintained behavioral goals (Braver, 2012), needs to be activated (Cohen et al., 1990). Indeed, the Stroop task, quoting Stroop in his original article (1935), is a measure of “interference of color stimuli upon reading words'' [p. 647] and is thus widely used to investigate both interference resolution (e.g., Nee et al., 2007) and selective attention, for which it is considered the “gold standard” (MacLeod, 1992). The ability tapped by the Stroop task allows us to selectively attend to specific properties in our environment based on our goals, while reducing the impact of potentially interfering information.

For several decades now, the Stroop task has been serving as a main tool for assessing executive attention disorders and impairments related to the frontal lobe, like anxiety, schizophrenia, dementia, and attention deficit hyperactivity disorder (e.g., Barkley, 1997; Henik & Salo, 2004; Spieler et al., 1996), for neuropsychological practice (e.g., Strauss et al., 2007), and in basic and applied research (e.g., MacLeod, 1992). For all that, many reviews have been conducted in several research fields (e.g., neuropsychology, Scarpina & Tagini, 2017; Periáñez et al., 2021; psychiatry, e.g., Peckham et al., 2010; Joyal et al., 2019; cognitive psychology, e.g., Algom & Chajut, 2019; Parris et al., 2022; Schmidt, 2019). Given this vast number of studies on the Stroop, including several important reviews, our intention of conducting a further review might not appear so straightforward. For this reason, in the next paragraph, we outline the goal of the present work to elucidate the contribution that we believe this review could give to the Stroop literature.

### Goal of the present review

Despite the plethora of studies and reviews on the Stroop task, consensus on many theoretical and methodological aspects is still far from being reached. For example, despite the Stroop effect often being regarded as a proxy for the activation of cognitive control mechanisms, some recent works questioned the validity of control-related and conflict adaptation explanations of it (see Algom & Chajut, 2019; Schmidt, 2019’s reviews as examples of two of the most influential ones). In addition to this example, a great variety of theoretical accounts have been put forward to explain the Stroop effect, some of which are in contrast to each other. Notwithstanding, it must be said that discussing such theoretical issues is not the aim of the present review, and we will present only the theoretical accounts useful for our purposes without going into much detail.

Here, we instead focus on the validity of the measures obtained with the Stroop task (Flake & Fried, 2020), that is, the extent to which the measures or results obtained using a research task or method actually represent the intended variable. Measurement validity indeed represents the fundamental requirement for any other form of validity, including the validity of the conclusions drawn from the experimental measures (Flake & Fried, 2020). However, since there are enormous methodological differences among studies employing the Stroop task and its adaptations, the validity of their Stroop measures is challenging to assess. In fact, these differences hinder direct comparisons between studies and, consequently, impede the possibility of drawing firm conclusions, potentially leading to inconsistencies at a theoretical level. For instance, it is emblematic that the Stroop task, along with other well-known experimental paradigms, exhibits the so-called reliability paradox, according to which the Stroop effect, despite being large, lacks reliability (Hedge et al., 2018; see also Viviani et al., 2023, for a more detailed discussion). The methodological confusion arises from the fact that, since the first study by Stroop, a multitude of Stroop task variants have been devised, often without relying on common guidelines. This is problematic because, as we will discuss in detail later, subsequent studies have highlighted the complex nature of the Stroop effect, demonstrating, for example, that it comprises multiple components (Parris et al., 2022). Therefore, it is crucial that, when we refer to the "Stroop effect”, all these components are taken into account. Despite the existence of works (e.g., Kornblum, 1992 discussed in detail later) that have explicitly clarified the necessary characteristics for a task to be considered similar to the classical Stroop and thus be called Stroop, such guidelines are commonly overlooked. Therefore, the message we aim to convey through this review is that methodological consistency among studies is essential whenever the label “Stroop task” is used to ensure a common ground. By claiming this, we mean that since the Stroop task originally proposed by its namesake author ensures a genuine and comprehensive Stroop effect, it represents the model to follow. Therefore, every replication of this task, both in terms of color–word versions and alternative variants, should strive to be methodologically similar to it, as only by using this approach, an accurate comparison of the evidence produced by individual studies is ensured.

The aim of the present work is to overview the tasks that have been denoted as Stroop tasks in the literature from a methodological point of view, to ascertain whether they can rightfully be called Stroop tasks, that is, if they are methodologically similar to the classical color–word version. However, by saying this, we absolutely do not mean that variations altering the classical version should be avoided altogether. On the contrary, if based on sound methodological assumptions, such variations can be useful, for instance, in gaining a better understanding of the nature of the Stroop effect or some of its underlying processes. Nonetheless, it is important that, when such variations do not adhere to the classical Stroop characteristics, they should not be labeled as Stroop tasks. Instead, it is preferable to use labels such as "Stroop-like" task to highlight this distinction and avoid the risk of misleading interpretations.

It should be noted that the methodological discussion in the present paper is not intended as a systematic review^1^ of the huge literature on the Stroop task and its alternative versions. Rather, the studies reviewed here must be considered just as examples of the main Stroop task versions serving our purposes of showing the methodological strengths and limitations of the general Stroop category they belong to. For this reason, our work is a narrative review that focuses on the specific methodological aspects we are interested in, to be informative and describe them, thus without specifically focusing on the selected exemplar studies (Uman, 2011). Therefore, we advise readers to consider this work from this perspective while ensuring that we have made every effort to avoid as much as possible any selection bias and to be as clear as possible.

Throughout this work, we will endeavor to demonstrate the reasons for our skepticism regarding the tasks commonly used in the literature, presenting several examples that highlight how the majority of Stroop tasks lack fundamental methodological requirements to be considered as such in all respects. At the same time, our objective is to encourage future studies to pay more attention to methodological aspects and the validity of Stroop effect measures. Nonetheless, our message is not to remain solely attached to the classical version of this task, which may present certain issues, such as the requirement for verbal responses that may not always be feasible, especially in experimental and neuroimaging settings. With a proactive intention, we thus propose an alternative family of Stroop tasks, the spatial variant, to demonstrate an example of an alternative Stroop version that ensures both methodological adequacy and, sometimes, greater flexibility. However, we wish to emphasize that our alternative proposal is not the only possibility, but merely one among many potential methodologically sound versions.

Given that we are not the first to propose a spatial version of the Stroop task and similar tasks have already been employed in the literature, a significant portion of this review will be dedicated to examining whether spatial Stroop tasks in the literature genuinely meet the criteria for being considered methodologically appropriate, that is, similar to the classical Stroop task. Nonetheless, before addressing the methodological requirements of the spatial Stroop task, we will provide an overview of the classical color–word Stroop task and its most popular variations.

Therefore, from a practical standpoint, the review is organized into two main sections. The first section outlines the necessary characteristics for methodologically sound Stroop tasks, followed by an overview of its most popular variants, providing examples to support our skepticism. The second section will focus on the spatial Stroop tasks found in the literature, assessing them, and explaining the reasons why we believe they may be a potentially preferable variant over many others.

### Object of our methodological inspection

The object of our methodological inspection is the Stroop effect as a whole. In the Introduction, we outlined two different approaches for calculating the Stroop effect, one contrasting incongruent and congruent trials, which allows obtaining only a global Stroop effect, and the other using also neutral trials, which allows portioning the Stroop effect into its facilitation and interference components. So far, it is not clear in the literature which of these two procedures is better to use. Indeed, the relative weights of interference and facilitation effects in composing the Stroop effect are currently unknown (MacLeod, 1991; MacLeod & MacDonald, 2000). In addition, whether interference and facilitation arise from a common mechanism (e.g., the congruency relationship between ink color and color name) or not is a further subject of controversy (e.g., Brown, 2011). Given these unresolved controversies, it seems more cautious to us not to distinguish between the two subcomponents. A further reason specifically regards the facilitation effect, whose reliability and stability have been called into question by findings showing that it is considerably smaller than the interference one, as shown by MacLeod & MacDonald's (2000) study, wherein facilitation effects were one-fifth the size of interference effects (for further evidence, see Augustinova et al., 2019; Lindsay & Jacoby, 1994). Additionally, Stroop facilitation measures have been shown to be affected by the baseline (i.e., the type of neutral trials) chosen to compute the contrast with congruent trials. Indeed, although colored non-words (e.g., XXX_green_) and color-neutral words (e.g., CHAIR_green_) are usually used interchangeably, converging evidence suggests that facilitation effects are underestimated when using colored non-words instead of color-neutral words (Augustinova et al., 2019; Brown, 2011). Of note, although it was not tested in the cited studies, the issue of baseline selection may also affect the comparison with incongruent stimuli and thus the calculation of Stroop interference, further supporting our choice not to distinguish between the two subcomponents of the Stroop effect. Finally, the facilitation effect still includes an interference component because a form of conflict occurs even on congruent trials. According to this view, since reading is assumed to be a more dominant and automatic process than identifying the ink color, even congruent trials are affected by a form of conflict, namely task conflict, and thus they cannot be considered as a pure measure of facilitation (Goldfarb & Henik, 2007; MacLeod & MacDonald, 2000; Parris et al., 2022). Indeed, task conflict in congruent trials is particularly evident in some cases (i.e., as a result of specific manipulations), as a phenomenon known as negative facilitation, characterized by longer RTs on congruent trials as compared to neutral ones, due to task conflict in the former but not in the latter (Parris et al., 2023).

Discussing the validity and the best methodological choices for measuring Stroop facilitation and Stroop interference separately goes beyond the scope of the present review, but we highlighted these issues to justify our choice to consider the Stroop effect as a whole, and not portioning it into these components. Moreover, the main reason for this decision is that our aim is to provide a methodological overview of the Stroop task that is as inclusive as possible; therefore, since most of the studies in the literature that used the Stroop task measured the Stroop effect and not its subcomponents, we decided to do the same. It is important to note, however, that most studies use the Stroop task to investigate processes similar to interference resolution, for which we are aware that the Stroop interference effect would be a much better and purer measure.

That being said, to avoid confusion, we will consistently use the generic term “Stroop effect” in the text, even when it would be more accurate to refer specifically to its interference component. We are aware that this may be a limitation, but we believe that it is the only way to ensure the generalization of what we discuss in this review. On the other hand, we believe that a methodical clarification regarding the facilitation-interference relationship would be necessary in the future to bring clarity to the matter.

## The color–word Stroop task: methodological considerations

This section is dedicated to some methodological considerations that we will consider as benchmarks throughout the entire review and, to justify their importance, we will draw on some theoretical accounts, which are required to understand the nature of the Stroop effect.

### Stroop effect asymmetry

The basis of the Stroop effect has been classically attributed to the so-called Stroop asymmetry, that is, the fact that task-irrelevant words slow color naming, while task-irrelevant colors interfere with word reading to a lesser extent (e.g., MacLeod, 1991; Melara & Algom, 2003). The prevalent explanation for this asymmetry is the automaticity account, according to which this occurs because the two dimensions imply different amounts of processing effort: naming ink colors requires more attentional resources than reading words, which is automatic and obligatory due to our extensive experience in reading and its consequent storage in procedural memory. Therefore, the more automatized process interferes with the less automatic one, but not vice versa (MacLeod, 1991). Based on these assumptions, the parallel distributed processing account of the Stroop effect (Cohen et al., 1990) is dominant in the literature and postulates that the Stroop asymmetry derives from the unintentional activation of the reading pathway, which is stronger than the weaker color naming one. Automaticity is thus considered on a continuum, relying on the strength of processing which, in turn, depends on the relative strength of the competing processes and can derive from several mechanisms (e.g., the effect of practice).

Alternatively, the commonly observed Stroop asymmetry has been explained by the dimensional discriminability account (Melara & Mounts, 1993), which posits that the relative speed of discrimination between the two dimensions, rather than the strength of processing, underlies such an effect because there is a mismatch in discriminability or salience between colors and words. Dimensional discriminability refers to the perceptual properties of the dimensions and, based on Melara & Algom's (2003) account, words are more discriminable than colors in most of the Stroop studies, explaining why they are processed faster and interfere more. Therefore, by matching the dimensional discriminability (e.g., by reducing the physical size of the words, making the colors more salient than the words, etc.) to render the dimensions equally discriminable, the Stroop effect can be deliberately reduced, eliminated, or even reversed (Algom & Chajut, 2019).

The dimensional discriminability account offers a reasonable explanation for the reverse Stroop effect which, as the name suggests, is produced when the typical Stroop asymmetry is reversed. This effect was first reported by Stroop (1935), who showed that after extensive practice in color naming, reading color words was impaired on incongruent trials. More recent evidence of color interfering with task-relevant word meaning was offered by Blais & Besner's (2007) study, in line with the dimensional discriminability account. In that study, when participants were required to identify a centrally presented colored word by pointing to the response word displayed in one of the four corners of the screen, the response latencies were longer when the target word appeared in an incongruent ink. The automaticity account provides a similar explanation for the reversed direction of interference, that is, it postulates that, if a normally more automatic process associated with one stimulus dimension is altered through radical experimental manipulations, such as those in the dimensional discriminability account, the normally less automatic process can become relatively more automatic, producing interference in the reverse direction (MacLeod, 1991). In other words, also according to the automaticity account, by changing the difficulty of processing, the interference can affect the process that should be stronger.

Evidence indeed exists in the literature supporting both accounts but, for the purposes of our review, they do not necessarily have to be considered as mutually exclusive. As such, we can speculate that the Stroop effect might be yielded both by differences in automaticity between the two dimensions and by differences in the discriminability of the two dimensions. When the discriminability of the two dimensions is the same, automaticity would be predominant, whereas when their processing automaticity is the same, discriminability would be predominant. With this in mind, researchers using the Stroop task should balance and control for both of them. For example, if automaticity is manipulated expecting that one dimension is more automatic relative to the other, the discriminability of the two dimensions needs to be controlled for. On the other hand, when discriminability is manipulated rendering one dimension more (e.g., perceptually) salient than the other, care must be taken to use equally strong processes.

To deliberately avoid favoring one account over the other, throughout this work, when we need to indicate that one dimension is prevailing over the other, we will use neither the term “more automatic” nor “more salient”, but we will neutrally refer to that dimension as stronger than the other.

### Stroop effect characteristics

The complex nature of the Stroop effect is not limited to the coexistence of interference and facilitation effects, but also extends to its composite nature. As such, in this section we provide a brief overview of the Stroop effect characteristics, with the aim of highlighting its fundamental requirements.

Over the years, a wealth of different single-stage theoretical accounts has been proposed to explain the nature of the Stroop effect; they can be divided into two general categories. The so-called late-selection accounts have been predominant in the Stroop literature and attribute the Stroop effect to response conflict, or interference^2^, in the response selection phase: in incongruent trials, the irrelevant word meaning elicits a (wrong) response that interferes with the selection of the correct response (Cohen et al., 1990; Posner & Snyder, 1975). In contrast, early selection accounts attribute the Stroop effect to stimulus conflict, suggesting that, in the stimulus-encoding stage, the irrelevant dimension of the incongruent stimulus interferes with the relevant one. According to some authors, stimulus conflict is perceptual in nature because it arises when colors are implicitly identified (e.g., Hock & Egeth, 1970), whereas others posit its conceptual and/or semantic nature and put forward that interference occurs because the meanings of both the word and color dimensions correspond to colors (e.g., Seymour, 1977). Within this early account framework, it has also been suggested that interference occurs at the task set level due to the conflict between the irrelevant but highly automatized task, that is, word reading, and the relevant task, that is, color naming (e.g., Augustinova et al., 2018; Goldfarb & Henik, 2006, 2007; Parris, 2014).

This distinction notwithstanding, it has been argued that the Stroop effect is better explained in terms of multiple-stage accounts, suggesting that processes at both the stimulus and response levels contribute to it. Stimulus- and response-based processes are not mutually exclusive but simply focus on different aspects of the Stroop task. Accordingly, Zhang and Kornblum (1998) examined stimulus–stimulus (e.g., between two incongruent stimulus dimensions) and stimulus–response (e.g., between two competing responses) effects both in isolation and in the Stroop task, showing that they interact in contributing to the Stroop effect. Their results suggest that the Stroop effect is due to the combination of stimulus–stimulus and stimulus–response compatibility (De Houwer, 2003; Zhang & Kornblum, 1998).

More recent studies further investigated the specific contribution of different types of conflict in the Stroop task, strengthening the evidence for an even more complex multiple-stage account. Augustinova et al. (2018) specifically tested the integrative assumption that the overall Stroop interference is composed of task, semantic, and response conflicts. To this aim, they compared response latencies to different stimuli, that is, standard color-incongruent words (e.g., BLUE_green_), associated color-incongruent words (e.g., SKY_green_), color-neutral words (e.g., DOG_green_), and color-neutral letter strings (e.g., XXXX_green_), and they then calculated the specific contribution of task (DOG_green_ – XXXX_green_ ), semantic (SKY_green_ – DOG_green_), and response (BLUE_green_ – SKY_green_) conflicts. When vocal responses were used, they clearly identified the behavioral signatures of each of these conflict types. Moreover, in a subsequent study, Augustinova et al. (2019) replicated these results and drew special attention to the effect of response modality, showing that the three conflict types contribute to Stroop interference only when vocal responses are used while, when manual ones are used, no task conflict is generated (see Section "Stroop stimuli and responses" for a more detailed discussion on this point).

In a similar vein, Parris et al. (2022) conducted a review to specifically investigate the processing levels that contribute to the Stroop effect^3^. They examined the evidence produced by studies in the literature to verify if it is consistent with the hypothesis that the Stroop effect is composed of multiple loci, which can be distinguished into informational and task loci. The former includes stimulus- and response-related conflicts/facilitations corresponding to previous single-stage models (e.g., De Houwer, 2003; Zhang & Kornblum, 1998), whereas the latter coincides with the above-described conflict between task sets. Their conclusions argue in favor of the multiple-stage account, suggesting that the Stroop effect arises at different loci. However, only two independent loci of attentional selection in the Stroop task were clearly differentiated; indeed, while there is evidence that task conflict is distinct from informational conflict, to date, measures distinguishing between stimulus and response conflicts/facilitations are still ambiguous. This notwithstanding, the authors left open the possibility of two distinct loci within the broader informational one but claimed the importance of developing more adequate models accounting for the multiplicity of Stroop loci. Additionally, neuroimaging evidence seems to point towards the same direction, as shown, for example, by the cascade of control model (Banich, 2009; 2019), which is an influential functional model accounting for Stroop performance in a multiple-stage manner.

Overall, although available findings are somewhat conflicting, evidence that the color–word Stroop effect occurs at different loci is convincing. As such, we can safely claim that, when designing a Stroop task, taking into account all these possible types (or loci) of the Stroop effect is of considerable importance. Therefore, to yield a Stroop effect involving all the three loci (which, for the sake of simplicity, we will call a complete Stroop effect), color–word Stroop tasks should entail: (1) interference at the task selection level due to conflict between two competing processing streams, naming and reading, with the former being less strong than the latter, (2) interference (and facilitation) at the stimulus processing level due to perceptual and/or semantic overlap between relevant and irrelevant stimulus representations, and (3) interference (and facilitation) at the response selection level due to the overlap between the two vocal responses activated by the ink color and the color name features (De Houwer, 2003; Funes et al., 2010).

As such, in the present review, the presence of three distinct loci will be evaluated in the overviewed studies. It is noteworthy that, while the presence of task conflict depends on the use of two distinct tasks activating competing task sets, the effects at the stimulus and response loci strongly rely on the characteristics of the Stroop stimuli and responses, which, therefore, should be designed carefully. In the next section, we focus on this latter point, discussing a relatively old account that, nonetheless, offers clear guidelines for designing Stroop tasks entailing both stimulus and response conflicts/facilitations.

### Stroop stimuli and responses

Both stimulus and response loci are incorporated in the dimensional overlap model put forward by Kornblum (1992) (see also Kornblum et al., 1990, 1999), which outlines the requirements that need to be satisfied to define a task as a Stroop task. This model is based on the concept of dimensional overlap, referred to as the degree of similarity, or correspondence, between two sets of items. Dimensional overlap can also be defined as the extent to which two sets of items have attributes or properties in common. It does not necessarily concern physical similarity, because it is a property of the representation of the sets and not necessarily a physical feature of them. Therefore, dimensional overlap can be perceptual and/or conceptual and can be observed in the stimulus and response sets, in two stimulus sets, or in both. The overlap can be measured on a continuous scale with different levels of similarity, going from totally dissimilar to totally similar. When two sets have dimensional overlap, taking one element from each set, they are either compatible if they match or incompatible if they do not match and interference is produced. Commonly, in the context of the Stroop task, to refer to the same concept, the term congruency, instead of compatibility, is used. Another key concept is the dimensional relevance, which concerns the degree to which the features of the stimulus are informative about the required response, which are defined as irrelevant when they are uninformative. Thus, relevant stimuli to which a participant is instructed to respond are distinguished from irrelevant ones, which should not be attended to but are usually difficult to ignore.

Combining dimensional overlap and dimensional relevance, Kornblum (1992) constructed a taxonomy to classify ensembles (i.e., the types of task) that produce compatibility effects, made of eight types of tasks characterized by increasing levels of dimensional overlap. At the first level, in the type-one ensemble, there is no dimensional overlap. At the opposite extreme, in the type-eight ensemble, there is dimensional overlap between all three task dimensions, namely, the relevant and irrelevant stimuli and the response dimensions. The color–word Stroop task is a typical example of this ensemble type, as there is dimensional overlap between (i) the irrelevant stimulus and response dimensions, (ii) the relevant stimulus and response dimensions, and (iii) the relevant and irrelevant stimulus dimensions.

According to this model, the response modality plays a key role in producing the Stroop effect, since the dimensional overlap between the stimulus and the response depends on this factor. Indeed, to produce interference/facilitation between stimulus and response, the type of response needs to overlap with the stimulus attributes. This implies that, in the color–word Stroop, naming (vocal) responses are needed to elicit interference/facilitation at the response level. Consistent with this, it has been shown that the color–word Stroop effect is considerably reduced with manual as opposed to vocal response modality, confirming the influence of the stimulus–response overlap (e.g., Augustinova et al., 2019; MacLeod, 1991). Nevertheless, the role of response modality remains a frequently ignored methodological issue and, in the literature, color–word Stroop tasks requiring manual (keypress) responses are commonly used (e.g., Ambrosini et al., 2019; Kinoshita et al., 2017; Szűcs & Soltész, 2010; Toth et al., 2019; Vallesi et al., 2017). However, according to Kornblum’s taxonomy (Kornblum, 1992), they cannot be considered as type-eight ensembles, or Stroop tasks, due to the lack of dimensional overlap between stimulus and response. Indeed, in Kornblum’s taxonomy, verbal Stroop tasks that require manual responses are classified as type-four ensemble and are referred to as Stroop-like tasks.

In the present work, the dimensional overlap model will be used along with the multiple loci account as benchmarks for evaluating the Stroop task methodology. In our opinion, both are useful for our purposes. This is because, while Kornblum’s account does not explicitly consider task conflict (but nonetheless all type-eight ensembles necessarily have task conflict), which is, however, mandatory to yield the Stroop effect, his taxonomy offers a clear framework for assessing in more detail the completeness of Stroop stimuli, especially for what concerns the suitability of the stimuli to produce the effect at the level of response. Indeed, the dimensional overlap model posits that two dimensional overlaps are required to produce a complete effect at the response locus.

### Confounding factors

In the previous section, we highlighted the methodological requirements for yielding a complete Stroop effect according to the multiple loci and dimensional overlap accounts, but they are not sufficient to ensure a methodologically sound Stroop task. Indeed, even when they are all satisfied, other factors might negatively affect the methodological quality of a task and the validity of the obtained measures, that is, confounders not related to the Stroop effect might bias its estimation, and thus they need to be controlled to ensure its validity. Therefore, in a methodological scrutiny, care must be taken to verify whether the task design allows excluding such confounding factors.

Among many possible confounders, one frequently encountered issue in the Stroop task literature regards the so-called priming or sequential effects, which refer to the fact that performance at the current trial (trial *n*) is influenced by the (partial or total) repetition of the characteristics of the preceding trial (trial *n*-1). Priming effects are also related to a conflict adaptation phenomenon, also known as the Gratton effect or the congruency sequence effect, which is commonly observed during the execution of the Stroop task. The Gratton effect refers to the fact that the congruency of the preceding trials influences the performance in the current one, with a Stroop effect that is smaller after incongruent trials and larger after congruent ones (Gratton et al., 1992; Kerns et al., 2004). Although it is generally recognized that conflict monitoring processes, mediated by a neural system including the anterior cingulate cortex and the lateral prefrontal cortex, are responsible for this phenomenon (e.g., Botvinick et al., 2001), some theories have provided alternative explanations for it. A detailed discussion of this effect is outside the scope of the present work, so the reader is referred to eminent works on this topic (e.g., Algom & Chajut, 2019; Algom et al., 2022; Schmidt, 2023). However, two main non-strategic explanations have been put forward to account for sequential effects, which both suggest that differences in RT between trial *n*-1 and trial *n* are not the result of cognitive control strategies, but exclusively an artifact of repetitions/alternations of either features or responses (Puccioni & Vallesi, 2012a, b, c). The former, known as the priming account, posits that the repetition of one or both stimulus features leads to facilitation effects, whereas the change of both features causes longer RTs (Mayr et al., 2003; Nieuwenhuis et al., 2006). The other explanation relies on the Theory of Event coding, which accounts for binding-type effects in object perception and action planning and proposes the existence of the so-called event files that temporally associate perceptual and action codes. During each Stroop trial, the stimulus and response features are linked in such event files. This gives rise to processing costs if in the next trial only some but not all features of such integrated codes are repeated, due to the need of file updating. Therefore, this theory predicts that performance is hampered if the feature match is only partial, a phenomenon known as the partial-repetition cost (Hommel, 2004; Hommel et al., 2004).

Although these two accounts predict different effects on performance, they both agree on the fact that the number of shared features between two subsequent trials can influence the Stroop and congruency sequence effects, which would thus be biased or confounded by such repetitions. Therefore, to have unbiased estimates of Stroop (and congruency sequence) effects, it is clearly necessary to design priming-free paradigms with a complete alternation in (at least) first-order trial sequences, that is, in the trial *n* both the relevant and irrelevant stimulus dimensions should be different as compared to the ones of trial *n*-1^4^. However, this cannot be achieved by using fewer than four possible irrelevant stimulus features and responses (i.e., the relevant stimulus features). For example, in a classical Stroop task, at least four color words in (the same) four ink colors should be used. Specifically, with three possible stimuli/responses, a repetition of either feature must inevitably occur in first-order trial sequence of two incongruent trials in a row (because each incongruent trial requires using two different features). Even worse, when using only two possible features/responses, only congruent-congruent sequences can be repetition-free, whereas this is unfeasible for any first-order trial sequence including an incongruent trial.

### Methodological standards

Throughout the entire work, thus, the tasks used in the studies we reviewed will be evaluated according to whether they are really suitable for measuring the Stroop effect as a whole (complete Stroop effect), as in the classical color–word Stroop task.

To this aim, as necessary standards of methodologically adequate Stroop tasks, we will use:

A) The multiple loci account for the Stroop effect to assess whether the obtained Stroop effect is comprehensive of the effects at the three processing loci detailed above;
B) Kornblum’s model to better evaluate the stimulus- and response-related processing levels assumed by multiple loci accounts. Specifically, we assessed whether the stimuli and responses employed ensure both stimulus–stimulus and stimulus–response overlaps.

Additionally, methodologically sound Stroop tasks should also employ experimental designs that allow controlling for confounding effects as much as possible (e.g., avoiding stimulus and response repetitions; see Section “Confounding factors”), to ensure measuring the Stroop effect with the necessary validity and reliability.

The importance of setting shared methodological standards arises from the great heterogeneity in the Stroop task literature. Indeed, under this umbrella term, several methodologically different experimental paradigms are included. Therefore, this confusion does not allow correctly interpreting their results, which are often conflicting, probably also due to the fact that the tasks actually measure partially, or even totally, different effects. The goal of the present review is thus to encourage the use of rigorous methodological criteria to design Stroop tasks, since starting from a common ground would allow having more adequate and valid Stroop effect measures.

The great heterogeneity in the Stroop literature also presented us with the need to use a unique inclusion criterion when selecting the studies, that is, the fact that the authors of the reviewed studies defined the task they used as a Stroop task. This general criterion is fundamental for our purposes of showing that, although all the tasks in the included studies were called “Stroop task” by the authors, many of them did not yield a complete Stroop effect.

## Other Stroop tasks

So far, we have been discussing some methodological aspects relevant to the Stroop task, specifically focusing on the color–word version. However, several alternative adaptations of the color–word Stroop task have been proposed. Thus, we will next present its most known and used variants, discussing them, and specifically considering the implications ensuing the methodological aspects we reviewed above.

### The picture–word Stroop task

The picture–word Stroop task, also known as picture–word interference task, is an alternative variant of the classical Stroop task. It typically consists of a word (distractor) printed inside a picture (target), which participants are asked to name (e.g., Arieh & Algom, 2002; Lupker, 1979; Rosinski et al., 1975)^5^. As such, conflict between task sets is usually present in this Stroop task category as in the classical Stroop task. Commonly, this Stroop variant is considered similar to the classical color–word Stroop task due to the presence of an asymmetry between words and pictures, that is, the interference is greater from word-to-picture naming than from picture-to-word reading (MacLeod, 1991; Rosinski et al., 1975). However, whether the word–picture interference effect is analogous to the Stroop effect is still a debated issue, as some authors claimed that they are caused by the same mechanisms (e.g., van Maanen et al., 2009), while others argued for their difference, suggesting that the picture–word interference effect occurs only at the level of perceptual encoding (Dell’Acqua et al., 2007). It is probable that these controversial results are due to differences in task design, that is, some experimental manipulations make it conceptually similar to the classical Stroop task, whereas others generate other types of effects. In what follows, we propose a distinction between the major experimental manipulations, proposing some specific labels which, in our view, could help reduce such heterogeneity. We would like to remind the reader that our aim is not to delve into the extensive and intricate literature on the picture–word Stroop task. Rather, our objective is solely to select specific studies as examples of the main manipulation types to examine whether they ensure a complete Stroop effect according to the criteria discussed above.

**Fig. 1:**
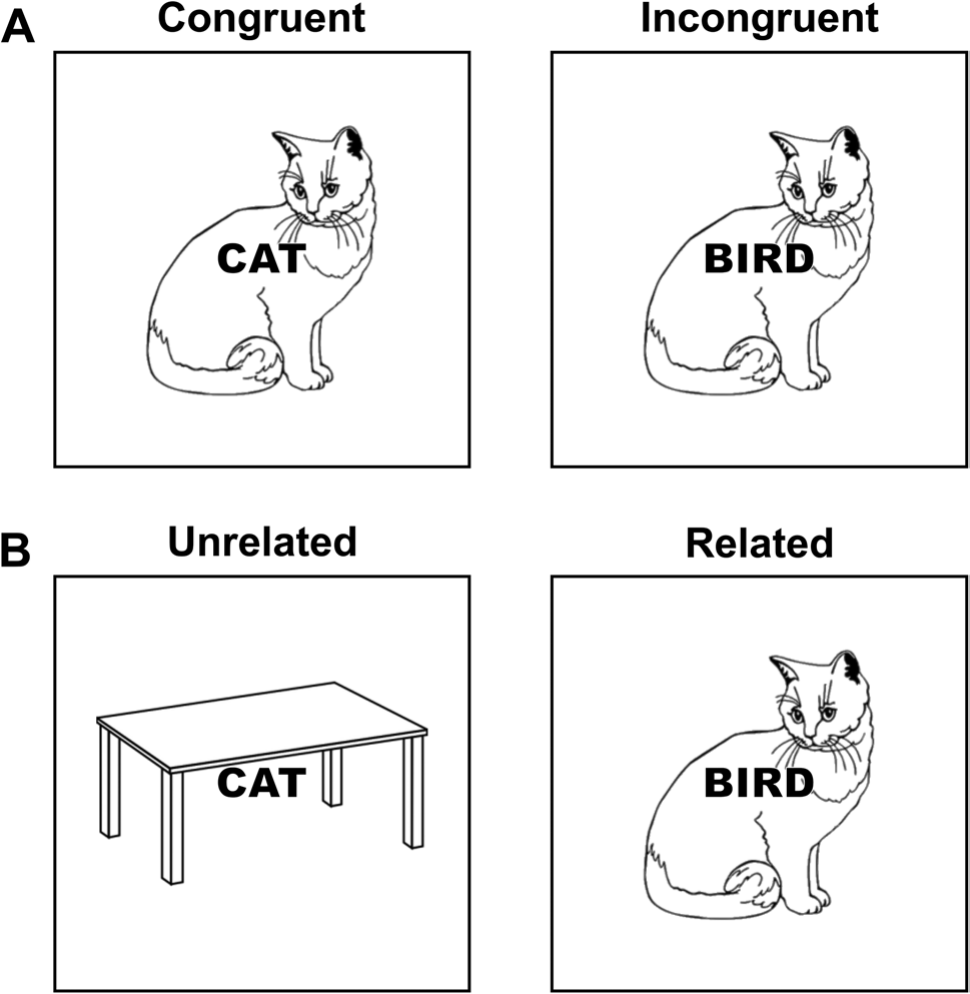
**A.** Example of the picture–word Stroop stimuli. Participants respond to the picture while ignoring the superimposed word. In the congruent condition, both the task-relevant and task-irrelevant stimuli refer to “cat”, while in the incongruent conditions, the picture (task-relevant) refers to a different item compared to the word (task-irrelevant), but both belong to the same semantic category. **B.** Example of the picture–word interference stimuli. Participants name the picture, ignoring the superimposed word. In the unrelated condition, the task-relevant stimulus is not related to the task-irrelevant one, generating less interference, while in the related condition, the semantic relation between the picture (task-relevant) and the word (task-irrelevant) produces interference

As outlined by Starreveld and La Heij (2017), semantic relevance, which is inversely related to the number of semantic categories from which the stimuli are selected, is important when comparing classical and picture–word Stroop tasks. Indeed, in the classical Stroop task, there is only one category, namely the color, whereas the number of semantic domains significantly varies across the existent picture–word interference experimental paradigms. This means that, when the picture–word Stroop task includes stimuli from the same semantic category, the produced effects can be equivalent or, at least, closer^6^ to the classical Stroop effect. Indeed, in this case, both congruent and incongruent conditions, analogous to the classical Stroop task, are possible: In congruent trials, the picture and the word refer to the same concept (e.g., the picture of a cat with the CAT word superimposed on it; see Fig. 1a), whereas in incongruent trials, they belong to the same semantic category but refer to different concepts (e.g., the picture of a cat with the BIRD word superimposed on it, which refers to another exemplar of the same semantic category of animals; see Fig. 1a), producing interference (e.g., Piai et al., 2013; van Maanen et al., 2009). As such, the picture–word Stroop effect, similar to the Stroop effect, can be calculated by comparing incongruent trials with congruent ones. For this reason, this version has been widely used in the literature investigating the Stroop effect. For example, Bugg et al. (2011) used a picture–word Stroop task in which words corresponding to one of four animal names were superimposed onto pictures of the same four possible referents. Thus, all stimuli belonged to the same semantic category (for a similar task, see also Gonthier et al., 2016). Moreover, usually in such picture–word Stroop tasks with a single semantic category, all task-irrelevant stimuli are eligible responses in the experiment, which is typical in the classical Stroop tasks. Lastly, according to the dimensional overlap model, picture–word Stroop tasks with one semantic category are classifiable as type-eight ensembles, that is, a Stroop task. Indeed, they ensure not only both stimulus–response overlaps, when using vocal responses, but also the stimulus–stimulus overlap because, although there are two spatially overlapped stimuli, they always have a non-negligible semantic relationship.

For such a similarity, henceforth, we will specifically refer to this version of the task with the label picture–word Stroop task (see Fig. 1a for an example), with the goal of distinguishing it from a conceptually different version, for which we will use the term picture–word interference task (Fig. 1b). In the latter case, the task is the same, but the stimuli are taken from different semantic categories, and there are no congruent trials (i.e., trials in which the picture and the word both indicate the same concept), but only trials with different degrees of picture–word semantic similarity^7^. Therefore, in this case the picture–word interference effect reflects the slowing in picture naming latencies when the picture is displayed with a conceptually related (but different) word – for example, the picture of a cat with the BIRD word superimposed onto it (see Fig. 1b) – relative to when the word is unrelated – for example, the picture of a table with the CAT word superimposed onto it (see Fig. 1b) (e.g., van Maanen et al., 2009; Shao et al., 2015).

The resulting picture–word interference effect was for long thought to originate during lexical access (the lexical selection by competition account, Roelofs, 1992; Vigliocco et al., 2004; see also the swinging lexical network account, Abdel Rahman and Melinger, 2009) or during other pre-response stages (i.e., perceptual encoding or activation of conceptual information, Dell’Acqua et al., 2007). So far the evidence is, however, scanty and contradictory (see Bürki et al., 2020 for a review). The underlying assumption is that when the task-irrelevant word is related to the target, its increased activation level yields competition for the lexical selection of the relevant picture, delaying its name retrieval during a naming task (e.g., Dell’Acqua et al., 2007; Roelofs, 1992). However, according to an alternative view, the response exclusion account, the semantic interference effect arises during the response execution (Mahon et al., 2007) and, thus, after the lexical access.

Therefore, the picture–word interference task produces a semantic interference effect that does not correspond to the one yielded by the classical Stroop task, also due to its semantic nature. Indeed, the semantic effect reflects selective inhibition, which is recruited when several responses are highly coactivated as part of the same response set, but it does not necessarily depend on the presence of an overt distractor stimulus. Therefore, semantic interference could be more similar to a semantic blocking effect, since both reflect selective semantic inhibition, rather than an effect yielded by interference resolution from an irrelevant distractor, such as the Stroop effect (Shao et al., 2015).

In addition to the difference in the underlying mechanism of the experimental effects we just discussed, two other main points have to be considered. First, in the picture–word interference task, the semantic relevance is lower compared to both the classical and the picture–word Stroop task because, to have unrelated trials, at least two semantic categories are typically used (e.g., objects and animals, as in Fig. 1b). Thus, this important component of the Stroop effect will be small or even absent if many stimuli taken from many different semantic categories are used (Starreveld & La Heij, 2017). The presence of several semantic categories also raises consequent issues related to the semantic gradient between the picture and the word, namely the semantic similarity between the picture and the word categories, which might be a confounding factor.

However, it is still controversial whether higher semantic similarity increases or decreases the experimental effect (Hutson & Damian, 2014), with some studies showing a greater effect for stimuli with greater semantic similarity (Vigliocco et al., 2004) and some others reporting the opposite pattern (Mahon et al., 2007). Therefore, it is not possible to choose semantic categories based on a reliable semantic gradient criterion to control for its confounding effect. A second fundamental difference is that the picture–word interference task involves a lower degree of response-set membership, since usually not all irrelevant words are part of the response set (Starreveld & La Heij, 2017). These methodological differences notwithstanding, the picture–word interference task can be classified as a type-eight ensemble, since there are both the stimulus–response overlaps, if the responses are vocal, and the stimulus–stimulus overlap whose degree, however, depends on the degree of semantic relevance/similarity.

Overall, if the aim is to investigate the Stroop effect, the picture–word Stroop task is preferable to the picture–word interference version, as the picture–word Stroop effect (congruent vs. incongruent) is a total Stroop effect as opposed to the picture–word interference effect (related vs. unrelated). However, although this experimental paradigm has the advantage of allowing flexibility in the stimulus set selection and in the possible manipulations (e.g., MacLeod, 1991), there are some potential issues to consider, which depend specifically on its linguistic nature. In fact, these issues also regard the classical Stroop task but, since the picture–word Stroop task allows one to select a potentially infinite set of stimuli, the matter is even more relevant for it. First, the same issues described above for the picture–word interference task, related to the degree of semantic similarity between the picture and the word, still apply to the picture–word Stroop task. However, this aspect is usually not explicitly controlled for in existing studies. Moreover, even if the vast majority of picture–word Stroop studies have focused on words belonging to the same category and sharing semantic features, many types of semantic relations affect word processing (e.g., De Deyne et al., 2019; Montefinese & Vinson, 2015), but their impact on picture–word Stroop effects is far from clear. For example, the few picture–word interference studies that have manipulated thematically and associatively related words (i.e., linked by a common situation or thematic context, but belonging to different semantic categories; e.g., the words COW and PASTURE) found no effect or even a naming facilitation (Abdel Rahman & Melinger, 2007; Alario et al., 2000; Costa et al., 2005; de Zubicaray et al., 2013), in contrast to the semantic interference effect robustly observed for categorically related words (e.g., the words COW and RAT), in which semantic similarity is usually derived from a feature production task (McRae et al., 2005; Montefinese et al., 2013).

Moreover, although semantic manipulations have the greatest influence on the Stroop effect, it can also be influenced by phonemic, graphemic, orthographic, and lexical manipulations. In fact, there is evidence that the orthographic and phonological aspects of the task-irrelevant stimuli and their relation with the task-relevant ones contribute to the Stroop effect (MacLeod, 1991). Therefore, if such linguistic components are not taken into account and balanced or controlled for when designing a picture–word Stroop task (which is a daunting task, due to the very complex interrelations between them), they might partly influence the magnitude of the Stroop effect. Moreover, the use of linguistic stimuli makes it very hard to adapt these tasks (and all the linguistic variants of the Stroop task) to different languages and, therefore, to generalize conclusions drawn from studies employing them.

### The numerical Stroop task

**Fig. 2:**
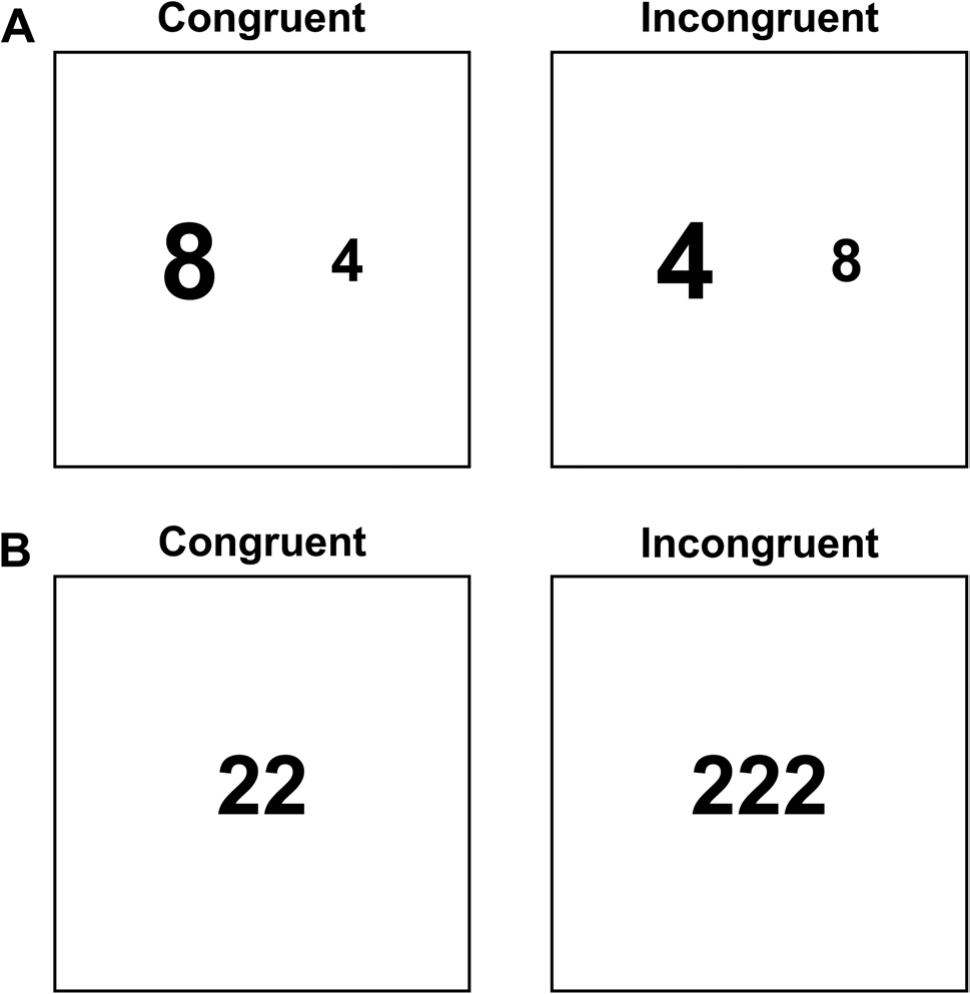
**A.** Example of the numerical Stroop stimuli. Participants identify the numerically larger digit, while ignoring its physical size. In the congruent condition, the numerically larger digit is also physically bigger, whereas in the incongruent condition, the magnitude (task-relevant) is in contrast with the digit size (task-irrelevant). **B.** Example of the counting Stroop stimuli. Participants indicate how many digits are displayed, ignoring the digit value. In the congruent condition, the digit quantity and the digit value are the same, while in the incongruent condition, the digit quantity (task-relevant) differs from the digit value (task-irrelevant)

In the numerical Stroop task (see Fig. 2a), firstly ideated by Henik and Tzelgov (1982), participants are presented with two Arabic digits, both of which are characterized by two dimensions: a physical one, namely font size, and a semantic one, namely numerical magnitude. The typical finding is that participants respond faster to numerically larger numerals appearing in a larger font size and to numerically smaller numerals appearing in a smaller font size (congruent trials) as compared to smaller numerals printed in a larger font size and to larger numerals printed in a smaller font size (incongruent trials), a phenomenon known as size congruity effect (SCE; Henik & Tzelgov, 1982). Findings regarding the asymmetry direction are mixed, as a congruency effect has been observed for both physical and numerical judgments. Indeed, it is commonly found that physical judgments are affected by task-irrelevant numerical distance, suggesting that numbers have a greater intrusive effect on size judgments than vice versa (Dadon & Henik, 2017). However, also numerical judgments have been shown to be influenced by task-irrelevant physical size (Borgmann et al., 2011). The fact that the SCE can be reversed has been accounted for in some works, which highlighted that the direction of the SCE asymmetry strongly relies on the discriminability of the dimensions and the number of employed values. Therefore, the greater intrusive strength of numbers would occur because commonly a higher amount of number values is presented (e.g., nine values, from 1 to 9), against few options of physical sizes (e.g., two, large vs. small). As mentioned earlier, since it is not our intention to delve into these controversies, we refer the reader to more specific works on this topic (e.g., see Algom et al., 1996; Pansky & Algom, 2002).

Therefore, moving beyond the issue of the asymmetry of the effect and its direction, the numerical Stroop task generally entails conflict between two different tasks, namely physical and semantic judgments. What is less certain is whether the dimensional overlaps at the other processing loci, namely stimulus and response ones, are ensured by numerical Stroop paradigms. According to Kornblum’s model, this task enables a dimensional overlap between the relevant and irrelevant stimulus dimensions. However, the stimulus attributes are relative and not absolute values, as they depend on the comparison between two different digit stimuli. Moreover, the so-called symbolic distance effect implies that the judgment speed is influenced by the numerical difference between the two numbers, with faster responses to larger differences (e.g., Tzelgov et al., 1992).

This potential reduction in stimulus–stimulus overlap is overcome by another Stroop adaptation that employs numbers as stimuli, the so-called counting Stroop task (Windes, 1968) (see Fig. 2b). The counting Stroop task typically consists of presenting numerals (e.g., 1, 2, and 3) in groups of different quantities (e.g., one, two, or three numerals). Participants are required to name the quantity of the numerals, while ignoring their value. The counting Stroop effect arises because, in incongruent trials, where the numeral and quantity do not match (e.g., three 2s are presented, see Fig. 2b), the response latencies are longer compared to congruent trials, where the numeral and quantity match (e.g., two 2s are presented, see Fig. 2b). This alternative version ensures not only an asymmetry effect, as naming the quantities of numerals is generally slower than naming the numerals (at least when their perceptual saliency is similar), but also a stimulus–stimulus overlap that more closely resembles the classical Stroop task one.

On the other hand, the presence of a stimulus–response overlap in both the numerical and the counting Stroop tasks deserves some specifications. First, only the counting Stroop task is suitable for vocal responses. When this response modality is used, despite the non-linguistic nature of the task, a stimulus–response overlap is ensured. Indeed, according to the Triple Code Model (TCM) of numerical cognition, numbers have three representational codes, namely Arabic, verbal, and analogical magnitude codes (Dehaene, 1992). Thus, in the counting Stroop task, the verbal code of the response overlaps with the codes of both stimulus attributes, namely, the relevant one referring to the magnitude code and the irrelevant one referring to the Arabic code.

Alternatively, manual responses can be used in both tasks, that is, in the counting Stroop participants can be instructed to use the corresponding keypress responses (e.g., pressing the 1 vs. 2 vs. 3 keyboard buttons to the corresponding numerical quantity), whereas in the numerical Stroop they can provide lateralized manual responses (e.g., left vs. right button press to smaller vs. larger numerical magnitude). Apparently, according to Korblum’s view, no stimulus–response overlap can be achieved when using manual responses, as in the counting Stroop there is no relation between the keypress buttons and either the numeral or the quantity, and in the numerical Stroop neither the physical size nor the numerical magnitude ensure such a relation. Nevertheless, in both tasks, the response might overlap with the stimulus if the response is considered to be compatible with the mental representation of magnitudes and numbers, based on the literature pointing out an association between space and numbers. Specifically, according to the Spatial-Numerical Association of Response Codes (SNARC) effect put forward by Dehaene et al. (1993), numerals are encoded and converted into magnitude representations and such magnitude information is organized spatially, for example as a mental number line, with smaller numbers on the left and larger numbers on the right (e.g., Montefinese & Semenza, 2018; Winter et al., 2015). Therefore, there would be a preferential stimulus–response association between smaller magnitudes and left-side responses and larger magnitudes and right-side responses (e.g., Dehaene et al., 1993). Therefore, if the response keys are spatially arranged consistently with the mental number line and/or lateralized to distinguish between smaller and larger magnitudes, the stimulus–response overlap would be warranted. However, such SNARC-related overlap might be weaker than the classical Stroop overlap and not universal, since it may be affected by cultural factors (i.e., the direction of reading and writing; see Dehaene et al., 1993; Zebian, 2005; also see Vallesi et al., 2014, for an analogue phenomenon in the spatio-temporal domain). It is also worth mentioning that a recent registered replication report (Colling et al., 2020) failed to replicate the attentional SNARC effect (Fischer et al., 2003), questioning the strong link between numbers and space.

In general, numerical and counting Stroop tasks could be advantageous for studying cognitive control from a more ecological and flexible point of view (Dadon & Henik, 2017) and to reduce the influence of linguistic factors while still ensuring task conflict. However, they also present some drawbacks, especially the numerical Stroop task. In fact, the counting Stroop task is a type-eight ensemble regardless of the SNARC-related interpretation, whereas the numerical one strongly relies on the SNARC to be considered as a Stroop task ensuring a stimulus–response overlap. Moreover, the experimental effects elicited by the numerical Stroop task might be affected by non-specific factors, such as the comparison time and the symbolic distance, making its measure less pure and threatening its measurement validity.

### The emotional Stroop task

**Fig. 3:**
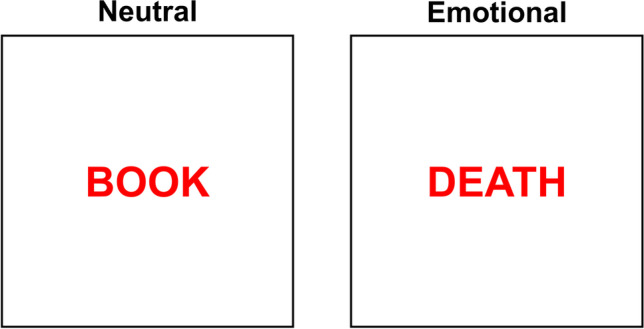
Example of the emotional Stroop stimuli: Participants are requested to name the ink color, while ignoring the word meaning. In the neutral condition, the word has a neutral meaning, while in the emotional condition, the word is emotionally charged but task-irrelevant

The emotional Stroop task (McKenna, 1986; Williams et al., 1996) (see Fig. 3) has been developed to examine attentional bias to emotional stimuli (Kappes & Bermeitinger, 2016). It requires participants to name the ink color of words, which can be either emotionally charged (e.g., the word DEATH), usually operationalized in terms of valence (i.e., the degree of pleasantness an individual feels toward a stimulus), or neutral (e.g., the word BOOK). In this task version, the interference effect is computed by subtracting the RTs to identify the color of neutral words from those to name the color of emotional words, also referred to as the emotional Stroop effect (e.g., Cothran & Larsen, 2008; Larsen et al., 2006). The underlying assumption is that the emotional content of the word interferes with color naming, causing longer RTs in identifying the color of emotional words compared to neutral ones (Wentura et al., 2000). This is again based on the assumption that, when participants are presented with isolated words, they cannot ignore their semantic meaning because they automatically access it (Larsen et al., 2006). Therefore, like in the color–word Stroop task, in the emotional Stroop task there is a task conflict between the stronger word reading and the less strong color naming (Cothran et al., 2012).

Still, the emotional Stroop task fundamentally differs from the classical Stroop task for several reasons. First, it lacks one of its fundamental properties, that is, the semantic relation between the relevant and irrelevant dimensions. More in detail, in the classical version of the Stroop task, the shared meaning of the compound stimuli allows manipulating the semantic congruency between such dimensions. In contrast, in the emotional Stroop task, there is no semantic or logical relationship between the relevant feature, namely the ink color, and the irrelevant feature, namely the emotional meaning of the word. This prevents generating congruent and incongruent trials and, consequently, the classical Stroop effect cannot be calculated (Algom et al., 2004; Cothran et al., 2012). The lack of semantic relationship between stimulus dimensions also entails no response-set membership because emotional words can never be part of the response set, thus no response conflict/facilitation can be produced.

Another critical aspect of the emotional Stroop task is the absence of lexical equivalence among emotional and neutral words. This represents a crucial difference from the color–word Stroop task, in which each word is presented in both congruent and incongruent conditions, causing an important methodological drawback. As such, Larsen et al. (2006) pointed out that the emotional Stroop is a quasi-experimental paradigm, because it does not allow having proper control conditions, as emotional words necessarily differ from control neutral words. Related to this point, there is still another difference from the classical Stroop task. Indeed, while in the classical paradigm the experimental effect can be calculated at the item level (i.e., for every color word), in the emotional version it can be measured only at the list-wide level: the absence of congruent and incongruent conditions allows only to compare mean RTs in naming the color of the emotional vs. neutral word lists (Algom et al., 2004; Larsen et al., 2006). Thus, the slowing of responses to emotional stimuli does not reflect a Stroop-like effect, but it could simply reflect a generic slowing to emotional stimuli. Specifically, Algom et al. (2004) argued for an automatic vigilance account for such an effect, showing that longer RTs to threat-related stimuli are not specific to the emotional Stroop task requiring ink color naming, but can be observed also in other tasks such as lexical decision tasks, reading speed, and word naming.

Finally, the emotional Stroop task does not allow controlling for possible confounding factors due to the linguistic nature of the stimuli. Indeed, what we highlighted regarding the potential role of confounding linguistic effects in the picture–word Stroop task also applies to the emotional Stroop task. Indeed, the emotional interference effect could also be affected by semantic, lexical, orthographic, or phonological factors, whose effect might be difficult to balance or control for. These aspects are more relevant for this task, compared to the color–word Stroop task, not only because it usually employs a much larger set of stimuli, but especially for the well-known differences that exist between emotional and neutral words, such as the fact that words with affective content generally are longer and have a smaller number of orthographic neighbors than the neutral ones (Larsen et al., 2006; see also Montefinese et al., 2013). Recently, in a multi-experiment study, Crossfield and Damian (2021) addressed this issue by matching neutral and emotionally valenced words for a number of lexico-semantic variables in an emotional Stroop task. The authors observed that the participants’ performance was mostly affected by semantic variables such as the word semantic diversity (i.e., a computationally derived measure of semantic ambiguity based on the variability of the different contexts in which a given word is used; Hoffman et al., 2013) and concreteness (i.e., the extent to which a word is related to sensorial experience), but also the word arousal (i.e., the degree of excitement or activation an individual feels toward a given stimulus, varying from calm to exciting). These results contribute to the longstanding debate on whether valence or arousal alone can produce the emotional Stroop effect. Moreover, they suggest that the valence effect is not powerful enough to generate the emotional Stroop effect by itself once other confounding variables are taken into account.

From Kornblum's model point of view, the lack of conceptual similarity between the relevant and irrelevant dimensions also implies the absence of a stimulus–stimulus overlap. Moreover, this task does not ensure any dimensional overlap between the irrelevant characteristic of the stimulus and the response, as neither vocal nor manual responses can overlap with the emotional meaning of the word. Therefore, the emotional Stroop is not even classifiable as a Stroop-like task.

In addition to the classical emotional Stroop variation, face-word Stroop tasks have been used in the literature as alternative emotional adaptations. Specifically, participants are presented with emotional words superimposed on faces whose emotional valence can be either congruent or incongruent with the word (e.g., the word HAPPY superimposed onto a face that expresses either happiness or sadness, respectively; Song et al., 2017). Since this experimental paradigm is an emotional form of the picture–word Stroop task, it suffers from the same issues as described above for that task, namely those related to lexical and semantic factors. Moreover, according to the dimensional overlap model, face-word Stroop tasks cannot be classified as type-eight ensembles because, although they ensure a stimulus–stimulus overlap, since the task-relevant and task-irrelevant stimuli have a relationship based on the presence vs. absence of emotion, they can never have a complete stimulus–response overlap because there is no face-response overlap, regardless of the type of responses used, that is, not even when vocal responses are used (assuming that emotion recognition does not necessarily activate lexical processing).

Overall, both versions of the emotional Stroop task are thus methodologically incomparable with the classical Stroop task. However, if the specific aim is to explore the effect of emotions on Stroop effect resolution, alternative emotional adaptations of the Stroop task may exist, such as the emotional priming Stroop task, in which an emotional vs. non-emotional prime stimulus is presented prior to a classical Stroop target stimulus. Recently, this experimental paradigm has allowed us to investigate how emotional processing affects conflict resolution, comparing neutral vs. sad face stimuli (see Visalli et al., 2022, for further details). This task represents a better alternative as compared to the other emotional Stroop tasks because, while the embedded Stroop task ensures all the required conflict types, including all the dimensional overlaps, it also allows exploring the influence of priming task-irrelevant emotional stimuli on the conflict/facilitation arising immediately after. The emotional priming Stroop task also overcomes another limitation of the emotional Stroop variants, namely lexical and semantic confounds, for example, by using faces or images as emotional priming stimuli, as in our recent study (Visalli et al., 2022).

### Other Stroop tasks: Conclusions

Taken together, it seems that the majority of the most known Stroop task variations present some theoretical and methodological issues, since they do not fulfill the criteria for yielding a Stroop effect comprehensive of the three required loci-related effects and/or are susceptible to potential confounding issues. Specifically, while it seems that the emotional Stroop and picture–word interference tasks tap on mechanisms that are different from the color–word Stroop task, this is not true for the other Stroop adaptations. In fact, the picture–word Stroop task entails both stimulus- and response-related interference/facilitation, as well as task interference, and has all the required overlaps, but the use of linguistic stimuli that are not only colors increases the possibility that confounding factors, such as the semantic gradient, influence the Stroop effect. This limitation can be overcome by the counting and numerical Stroop tasks, but their Stroop effect, in turn, might be affected by symbolic distance. Moreover, these tasks, especially the numerical Stroop one, have a SNARC-related stimulus–response overlap. Hence, although these alternative versions have some advantages for specific research topics, using the label “Stroop task” for them is, in some cases, inappropriate from a methodological point of view, as the experimental effects they produce rely on totally or partially different mechanisms. Moreover, even those alternative versions that ensure a complete Stroop effect have some drawbacks that potentially affect it and are difficult to control.

Since our aim is to highlight the importance of methodological rigor when designing Stroop tasks to ensure complete Stroop effects with the required measurement validity, the previously discussed alternative versions seem less adequate. For this reason, here we propose the spatial Stroop task as an alternative Stroop adaptation that potentially meets the required criteria and overcomes the drawbacks of the other versions. In the next section, we discuss this task in more detail, highlighting why it might be preferable compared to the other task versions reviewed above.

### The spatial Stroop task

The spatial Stroop task explores the interference/facilitation produced by irrelevant spatial information. Typically, verbal or symbolic stimuli are used to combine a semantic attribute indicating a spatial location with an attribute designating a physical position. As in the classical version, the stimuli can be either congruent or incongruent, depending on whether the physical position corresponds or not with the semantic attribute, producing interference in the second case (see Lu & Proctor, 1995, for an overview). For example, when a location word (i.e., LEFT, the semantic attribute) is presented in an incongruent physical position (i.e., right), RTs are longer compared to congruent conditions (i.e., LEFT presented at the left location) (Pang et al., 2020). From this general definition, it can be noticed that the term spatial Stroop does not necessarily refer to a purely spatial version of the Stroop task, as sometimes verbal stimuli, despite referring to spatial attributes, are employed. However, purely spatial versions of this task can be designed, for example by replacing location words with non-verbal semantic attributes, such as arrows. Later in this section we will provide some examples of pure spatial Stroop versions and we will discuss why such pure variants are preferable.

Before that, we will point out why, potentially, the spatial Stroop task can be considered as a methodologically valid alternative version of the Stroop task. To this end, we will use as an example a simple version of a purely spatial paradigm, wherein participants use right-left keypresses to respond to the direction of an arrow (i.e., right- vs. left-pointing) while ignoring the position on the screen where it appears (i.e., in the right or in the left side of the screen). The arrow direction is the relevant information, whereas its position is the irrelevant one. This task works exactly as the classical Stroop task, as it also entails an asymmetric relation between the stimulus dimensions (Lu & Proctor, 1995) and yields a conflict between two competing task sets. Indeed, it is assumed that the position of a visual stimulus is processed more strongly than its other visual characteristics, such as the pointing direction of an arrow (Viviani et al., 2023). In addition to task conflict, this task entails both stimulus- and response-related conflicts/facilitations and, according to Kornblum’s dimensional overlap model, it can be classified as a type-eight ensemble. The three overlaps are indeed all present and, specifically, they can be observed between: (i) the relevant stimulus attribute (direction) and the irrelevant one (position), (ii) the relevant stimulus attribute and the response dimensions, and (iii) the irrelevant stimulus attribute and the response dimensions^8^. The presence of a dimensional overlap between the stimulus dimensions and the response, namely the last two criteria, is strictly related to the response modality, as discussed above. Indeed, to obtain stimulus–response compatibility, responses need to overlap with irrelevant and relevant stimulus attributes. In purely spatial Stroop versions, as in the case of our example, the spatial arrangement of the keypress responses overlaps with each stimulus dimension due to the spatial nature of both the relevant and irrelevant stimulus attributes. By contrast, had the task been designed with vocal responses, or even with non-overlapping manual responses, such overlap would not have been possible.

Overall, the possibility of obtaining a complete Stroop effect with the spatial Stroop task suggests that it represents a valid adaptation of the classical paradigm. Besides being methodologically similar, the spatial Stroop version offers some advantages over the classical vocal Stroop task. The three main advantages are: (i) it does not rely on linguistic processing, as discussed above, (ii) the spatial nature of the stimuli might foster a more domain-general investigation of cognitive control, minimizing the confounding role of linguistic demands, and (iii) it requires manual responses, which are less prone to assessment errors and more suitable for neuroimaging and online studies than verbal responses (for a more detailed discussion, see Viviani et al., 2023).

Taken together, these advantages are related to the fact that the classical Stroop is in general more complex and prone to confounding. Indeed, as discussed above, due to its linguistic nature, the produced interference/facilitation effects might be influenced not only by the semantic relationship between the relevant and irrelevant dimensions, but also by a number of other linguistic variables, which are related to each other in such a complex way that it is very hard to control for them appropriately. Moreover, as we have already mentioned, the use of linguistic materials makes it harder to adapt these variants to other languages and limits the generalizability of the obtained results and conclusions. For these reasons, the spatial variant represents a valid alternative to bypass these potential drawbacks.

However, it is worthwhile noting that these advantages hold specifically for purely spatial paradigms because they are the only adaptations in which a complete Stroop effect can be elicited. Indeed, in spatial Stroop paradigms comprising both verbal and spatial stimuli, the choice of response modality is more problematic, since there is evidence suggesting that keypress responses to word meaning are affected by irrelevant stimulus position, whereas the interference is much smaller in the opposite direction; moreover, vocal responses to location are influenced by irrelevant word meaning, but not vice versa (Lu & Proctor, 1995). However, notwithstanding the correct response modality being employed, in the mixed versions, the dimensional overlap is necessarily limited to either relevant stimulus and response or irrelevant stimulus and response dimensions, whereas both these overlaps are not simultaneously achievable. Moreover, spatial paradigms with verbal stimuli do not allow a complete reduction of the involvement of linguistic processing, with the consequent limitations outlined above.

In the next section, we shall present an overview of the spatial Stroop tasks that have been used in the literature. Of note, our work does not intend to be a systematic review of the literature on the spatial Stroop task, but has the explicit aim of focusing on the methodology of these paradigms. For this reason, we will not discuss the results of such studies in detail.

## The spatial Stroop task in the literature

Our search for spatial Stroop studies showed that there is a great variety of task versions, as a plethora of stimulus types and manipulations have been used. Hence, to make our discussion more systematic, we will distinguish three categories among which, in our view, only the third one has the potential to yield a complete spatial Stroop effect: position–word, arrow–word, and arrow–position tasks.

### Position–word spatial Stroop tasks

**Fig. 4:**
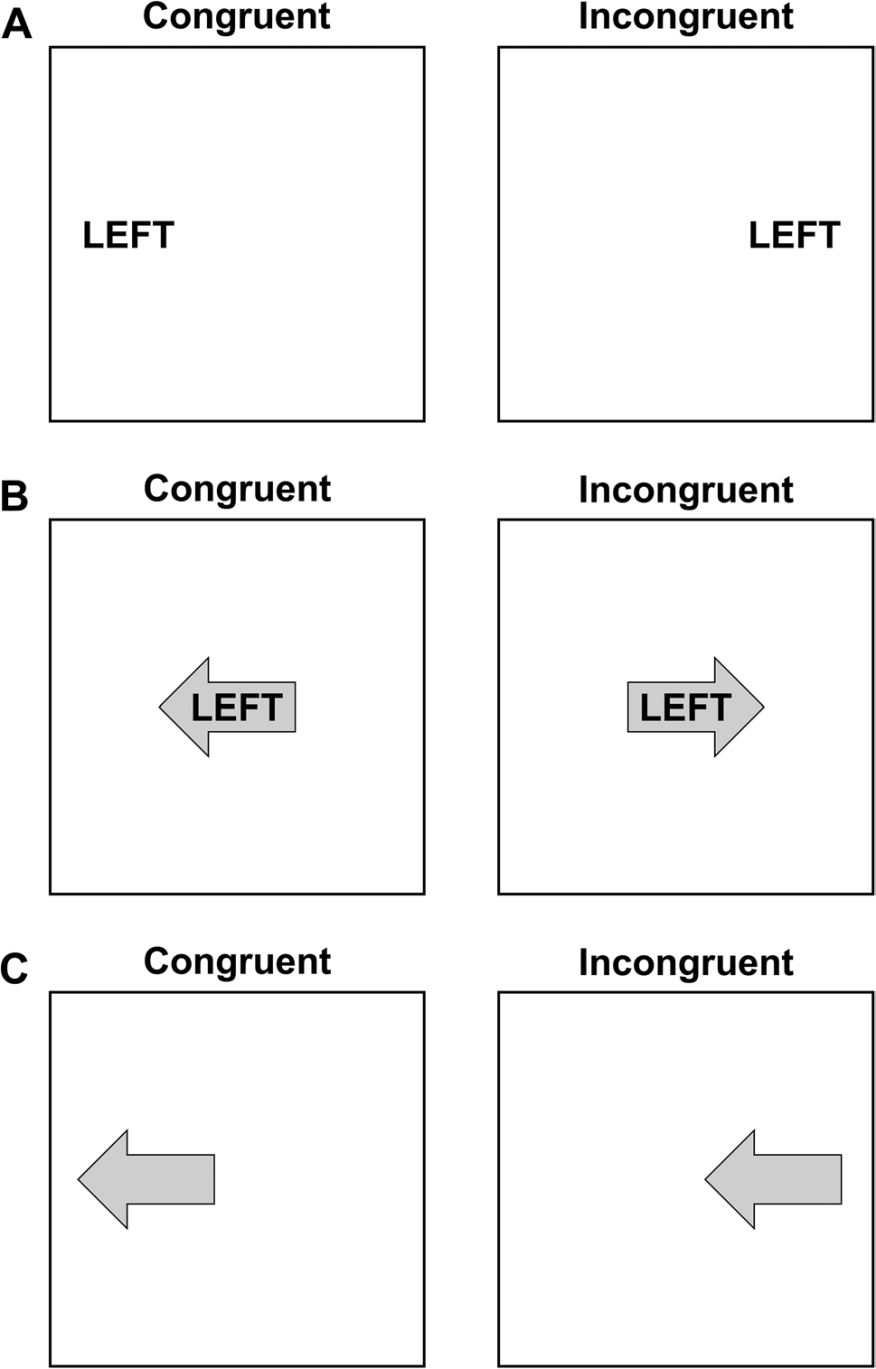
**A.** Example of the position–word spatial Stroop stimuli. Participants identify the physical position on the screen of a word designating a spatial location (i.e., LEFT word). In the congruent condition, the spatial location word is presented in the same physical position it denotes, whereas in the incongruent condition, the spatial location word (task-relevant) is in contrast with the physical position (task-irrelevant) where it appears. The task can also be reversed, that is, participants name the physical position of the word, ignoring its meaning. In this case, the task-relevant feature is the physical position, whereas the task-irrelevant feature is the spatial location designated by the word. **B.** Example of the arrow–word spatial Stroop stimuli. Participants name the direction of the arrow (i.e., left), ignoring the meaning of the spatial word printed inside. In the congruent condition, the arrow points toward the same spatial position designated by the word, whereas in the incongruent condition, the arrow direction (task-relevant) is in contrast with the spatial location denoted by the word. The task can also be reversed. **C.** Example of the arrow-position spatial Stroop stimuli. Participants identify the direction pointed by the arrow (i.e., left), ignoring the position where the arrow appears. In the congruent condition, the arrow points to the same direction as its physical position, whereas in the incongruent condition, the arrow direction (task-relevant) is in contrast with its physical position (task-irrelevant)

In position–word tasks, words designating spatial locations are displayed in congruent or incongruent positions on the screen ﻿(see Fig. 4a). It is noteworthy that in the literature words and positions are usually both considered to be processed in an equally strong way (at least when their perceptual saliency is kept similar). Therefore, when using them together, an asymmetry effect seems less likely as, at least in principle, none of the two tasks appears obviously stronger than the other. The lack of such an asymmetry can be noticed in the studies we will discuss, as some require word reading, while others position naming. Of note, being typically equal the strength of these two tasks, the spatial Stroop effects might also be driven by the effect of discriminability (as discussed above and as predicted by Algom & Chajut, 2019).

Some of the first studies using the spatial Stroop belong to this category, such as the one conducted by White (1969), in which the words NORTH, SOUTH, EAST, and WEST could appear in one of these four positions, and participants had to vocally indicate the position of the word, while ignoring its meaning. Therefore, besides the stimulus-related effects due to the stimulus–stimulus overlap and the task-related conflict, this task has an irrelevant stimulus–response overlap but not a relevant stimulus–response overlap; thus, it still does not produce full response-related effects.

In a more recent study, Luo and Proctor (2013) designed three spatial Stroop paradigms, all of which had in common that both relevant and irrelevant stimulus dimensions referred to “up” or “down” attributes, and participants were instructed to respond using bimanual right-left keypresses to ensure that responses were orthogonal to the relevant stimulus dimension. In Experiment 1, they presented the Chinese characters for UP and DOWN, appearing above or below on the screen. Participants were first asked to respond to the word and, in a second session, to respond to its location. Overall, regardless of their type, the stimuli did not overlap with the response, thus hindering any conflict/facilitation emergence at the level of response. However, both task conflict and stimulus–stimulus overlap were ensured. The other task variations used in that study (Luo & Proctor, 2013) will be discussed in the arrow–word spatial Stroop task section.

In Hilbert and colleagues' study (2014), participants were presented with four squares in the upper, lower, right, or left portions of the screen. The German words for UP, DOWN, RIGHT, and LEFT appeared within one of these four squares and participants were instructed to respond to the square position independently of the word meaning. All participants performed both an analog version of the task, in which they responded verbally, and a digital version, wherein they used four spatially arranged keypresses as the four locations. Therefore, in the analog version, the relevant stimulus did not overlap with the response, whereas in the digital version, the irrelevant stimulus did not overlap with the response. Thus, in neither case, conflict/facilitation effects at the level of response locus could be ensured. Nevertheless, the resultant Stroop effect included conflict at the task level and stimulus-related interference/facilitation.

Pickel et al. (2019) presented participants with the same four direction words (UP, DOWN, RIGHT, LEFT), but they could appear only in two possible locations, each obtained from a mix between two physical positions in space (upper right or lower left). Button presses were used for responses to word meaning, thus allowing an overlap only between the irrelevant stimulus and the response. Moreover, this paradigm had a lower degree of overlap between relevant and irrelevant stimulus dimensions because there were four relevant stimuli but only two irrelevant dimensions, not ensuring full experimental effects at the stimulus level. Thus, in this study, the Stroop effect was driven mainly by the task conflict.

One last example of this category is the study conducted by Schneider (2020), who designed two position–word spatial Stroop tasks. One required participants to respond to the words RIGHT and LEFT, appearing on the right or left of the fixation, while the other required participants to respond to the words UP and DOWN, positioned above or below the fixation. Notably, in both paradigms, responses were bimanual and were made by pressing spatially compatible keys, ensuring also an irrelevant stimulus–response overlap. Thus, the effect of conflict/facilitation was produced at the stimulus locus but not completely at the response locus, missing the relevant stimulus–response overlap. Although task conflict was also ensured, this paradigm did not produce a complete Stroop effect, besides not being priming-free as only two stimuli were employed (i.e., feature repetitions could not be avoided).

### Arrow–word spatial Stroop tasks

Arrow–word tasks entail presenting words referring to directions, embedded in or flanked by arrows (see Fig. 4b). Differently from position–word arrow Stroop tasks, an asymmetrical relation is possible, as word reading may be conceivably assumed to be stronger than direction identification. Despite that, in the following studies, word reading was not always a task-irrelevant process, and this could be interesting for further investigating the actual existence of such an asymmetry.

One of the first examples is the study conducted by Shor (1970), in which the word names of directions (UP, DOWN, RIGHT, LEFT) were embedded in arrows pointing to these four directions. The task was first to name the arrow direction and then to read the words, guaranteeing in both cases a conflict between task sets. The asymmetry was confirmed, as the naming of arrow directions was slower than the reading of words. Furthermore, this task design ensured the experimental effects at the stimulus locus, thanks to the stimulus–stimulus overlap, but not at the response locus, as the stimulus–response overlap was not complete and depended on the task at hand (i.e., when the task was direction naming, there was an overlap between the irrelevant stimulus and the response, and when the task was word reading, there was an overlap between the relevant stimulus and the response).

Luo and Proctor's (2013) study, which was already introduced in the position–word spatial Stroop task section, included two more experiments, wherein the same direction words (Chinese words for UP and DOWN) were either embedded in an up- or down-pointing arrow (Experiment 2) or flanked by an up- or down-pointing arrow (Experiment 3). Participants again underwent two sessions, responding to the direction word and then to the arrow direction. The major drawback of these two tasks was the same as the position–word one, that is, the total absence of response-related conflict/facilitation, as there was no stimulus–response overlap, due to the orthogonality of the right-left keypress responses.

A very similar study was conducted by Pang et al. (2020). To investigate global precedence, they presented participants with Chinese characters (UP, DOWN) embedded in up- or down-pointing arrows and asked them to respond to the character meaning or to the arrow direction by means of right-left keypress responses. In a second experiment, they reversed the stimuli, embedding the arrows in the Chinese characters. Again, according to our view, the Stroop effect was not complete, as by lacking stimulus–response overlap, it did not ensure producing experimental effects at the response locus.

### Arrow-position spatial Stroop tasks

In arrow-position tasks, participants are instructed to respond to the direction of an arrow regardless of its position on the screen (e.g., Pires et al., 2018; see Fig. 4c). In this task, there is an asymmetry between position and direction, as the former task is stronger than the latter.

This variant was used by Funes et al. (2007) in a paradigm combining a spatial Stroop task with spatial cueing. The spatial Stroop paradigm consisted of responding to the direction of right-/left-pointing arrows appearing either at the right or at the left of a fixation cross. Among the several experimental manipulations, of interest here is the one regarding response compatibility. More in detail, keypress responses were spatially compatible (e.g., left key for left direction) or incompatible, when the opposite response mapping was used. According to Kornblum’s model, only the former case ensures stimulus–response overlap, since the spatial arrangement of the response keys was compatible with the arrow directions and, as a consequence, full response-related effects were generated. In contrast, when the response keys were incompatible, there was no overlap between stimulus and response and no effects at the response locus. In both cases, both task conflict and stimulus-related conflict/facilitation were guaranteed, but priming effects could not be ruled out.

Luo et al. (2010) presented up-/down-pointing arrows positioned along the vertical axis and used bimanual right-left keys for responses. Although their aim was to have a pure measure of the Stroop effect, the response key spatial arrangement did not allow for a stimulus–response overlap, and consequently a complete Stroop effect, since it ensured only task conflict and stimulus-related conflict/facilitation.

In Pires et al. (2018) study, participants responded to right-left arrow directions, appearing in one of two lateral boxes (on the right or left of a central box). Responses were made using bimanual right-left button presses. Therefore, according to our methodological criteria, this paradigm produced a Stroop effect comprehensive of all required loci: task conflict, stimulus-related effects due to stimulus–stimulus overlap, and response-related effects due to stimulus–response overlaps. However, the use of only two characteristics makes it vulnerable to priming effects.

In a very recent study, Paap et al. (2020) used two versions of the spatial Stroop. The first version was a horizontal arrow Stroop task, with right and left arrows displayed on the right or left of the fixation, whereas the second one was a vertical arrow Stroop task, with up and down arrows presented either above or below the fixation. During both tasks, participants were instructed to respond to the direction of the arrow, using bimanual right-left keypress responses and ignoring position, generating task-related conflict. As highlighted by the authors, the horizontal task ensured both stimulus–stimulus and stimulus–response overlaps, and thus all the three processing loci, while the vertical version generated an overlap only at stimulus–stimulus level, but not at stimulus–response level, as the response keys were orthogonal to the direction of the arrows, impeding the effects to emerge at the response level.

In the same study discussed previously (see position–word spatial Stroop task section), Schneider (2020) proposed two versions of an arrow-position spatial Stroop task, one with a horizontal alignment and another with a vertical alignment. In the former, participants were instructed to indicate the direction of right-/left-pointing arrows appearing either on the right or the left of the fixation, whereas in the latter they responded to the direction of up-/down-pointing arrows appearing either above or below the fixation. Bimanual responses were made using compatible keypresses, as they were located as a function of the stimulus spatial alignment, thus having the potential to produce in both tasks not only stimulus–stimulus overlap and stimulus-related effects, but also stimulus–response overlaps and complete response-related effects.

Lastly, a very recent study by Spinelli et al. (2022) explored conflict adaptation using the color–word task (Experiment 1) and the arrow-position Stroop task (Experiment 2). In the spatial Stroop task, participants responded to one of six possible arrow directions (north-east, east, south-east, south-west, west, north-west), which could appear in one of six circles spatially arranged in the same six locations. Responses were made using keypress buttons whose spatial arrangement was compatible with arrow directions and positions. Hence, this experimental paradigm ensured all the conflicts/facilitations assumed by multiple loci accounts, that is, task conflict, stimulus-related effects due to the stimulus–stimulus overlap, and complete response-related effects due to the stimulus–response overlaps, despite a complex 6 x 6 stimulus–response mapping.

### Methodological considerations

**Fig. 5:**
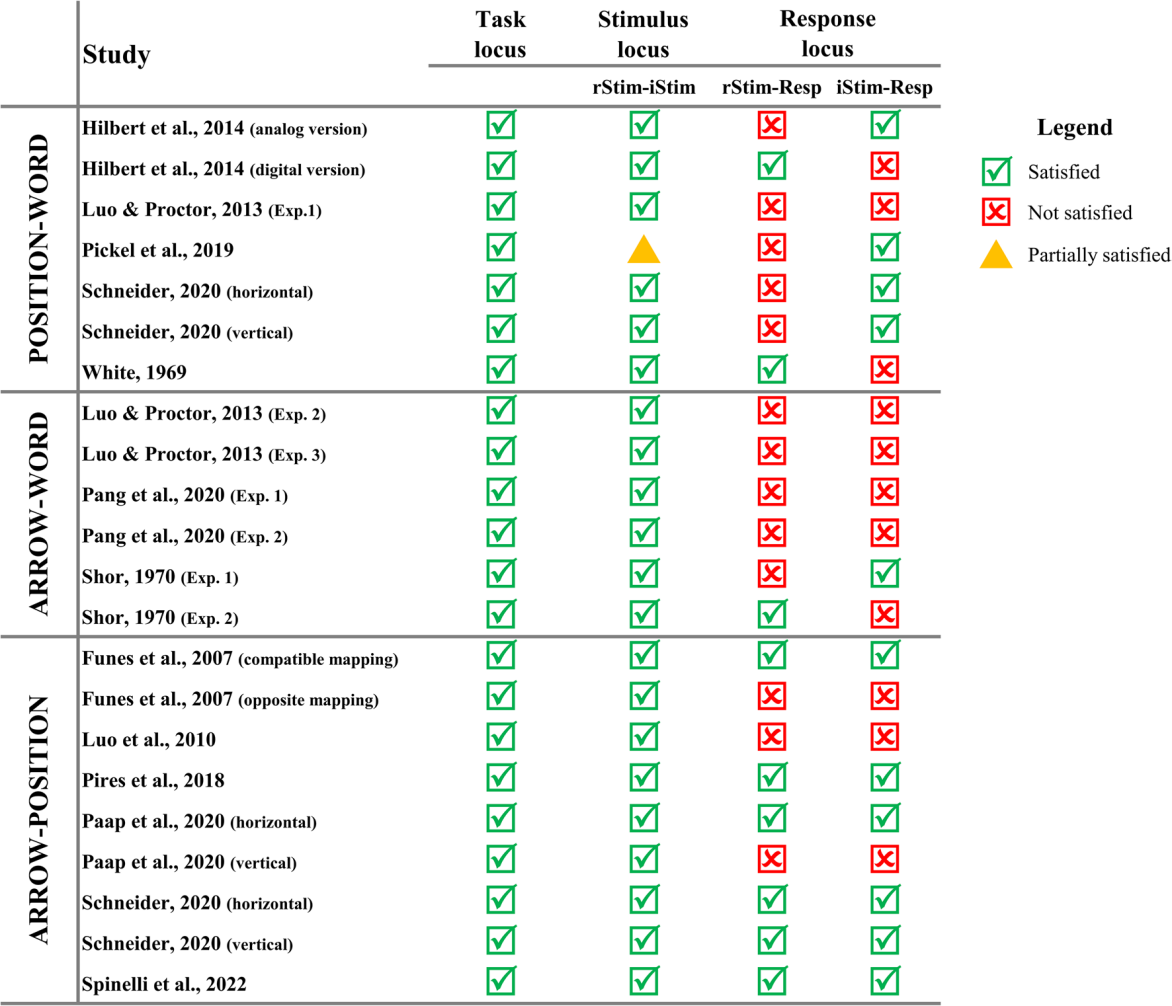
Summary of the methodological criteria met by each spatial Stroop task, also including Kornblum’s overlap levels. *rStim* task-relevant stimulus feature, *iStim* task-irrelevant stimulus feature, *Resp* response

From this methodological review, the first aspect worthy of consideration is that spatial Stroop paradigms involving only effects at the task and stimulus levels, without the involvement of response locus due to totally or partially missing stimulus–response overlaps, seem quite common (see Fig. 5). Indeed, several authors deliberately declared to use only stimulus–stimulus congruency to have more pure spatial Stroop effects, and explicitly distinguished it from stimulus–response congruency, regarded as a possible confound and/or as typical of just the Simon congruency effect (e.g., Funes et al., 2010; Luo et al., 2010, 2013). However, as highlighted above, both stimulus–stimulus and stimulus–response overlaps are required to obtain a complete spatial Stroop effect. Designing tasks to measure the Stroop effects in a methodologically rigorous way is not an end in itself but is of fundamental importance to measurement validity (and all the other forms of validity that depend on it) and, in turn, the improvement of Stroop measure validity is essential to enhance our theoretical knowledge about cognitive processes involved in the Stroop task. Indeed, the use of experimental paradigms that only tap on some mechanisms and ignore others, such as those that measure only task-related and stimulus-related effects, is inconsistent with the goal of obtaining an accurate measure of the Stroop effect because they do not consider the response locus, which is instead involved in Stroop tasks. Of course, if the aim of the study is to explicitly focus on one of the underlying mechanisms, this is warranted, but this has to be clearly stated (and in this case the label Stroop-like task is preferable). Moreover, the use of heterogeneous tasks does not allow us to compare their results across studies because, if these tasks are inherently different, they inevitably measure different phenomena. Thus, given that the Stroop effect includes multiple loci, that is, it involves processing at the level of task, stimulus, and response loci, it is clear that one needs to design tasks encompassing all these loci to obtain a measure as complete as possible.

Secondly, as we previously foreshadowed, position–word and arrow–word spatial Stroop tasks are not ideal versions of this paradigm. Besides not being pure spatial Stroop tasks, some of these mixed variants were not Stroop tasks due to the response modality employed. Since the presence of response effects depends on it, the response modality when the irrelevant stimulus was spatial should have been distinguished from when it was verbal. However, this was not the case, as the majority of studies entailed manual responses, regardless of these theoretical considerations. However, although this assumption was met, the simultaneous presence of a verbal and spatial stimulus would prevent a complete overlap between the stimulus and the response, as the response could overlap only with either the irrelevant or the relevant stimulus, but not with both at the same time, consequently hindering a full conflict at the level of response.

Therefore, in our view, the spatial Stroop tasks that most adhere methodologically to a complete Stroop task definition are the purely spatial ones, such as the arrow-position tasks. Indeed, from a methodological point of view, they are preferable because they potentially guarantee the possibility to produce effects at all the three required loci, ensuring all Kornblum’s dimensional overlaps. However, this was not true for a minority of arrow-position tasks which, totally or partially, did not involve the response locus (e.g., Luo et al., 2010).

In addition to these considerations, the present literature overview allowed us to notice a further methodological limitation concerning all of the three categories, which should be taken into account when designing a spatial Stroop task. This limitation, specifically, is that the majority of the studies used two-alternative forced-choice tasks, that is, in most of them the relevant/irrelevant dimensions were right vs. left or up vs. down, but rarely more stimuli and responses were used in the same task. The oldest paradigms (Shor, 1970; White, 1969) and few more studies (e.g., Hilbert et al., 2014; Pickel et al., 2019) were exceptions. This is a kind of pitfall, as it poses limitations in the manipulation of the trial list sequence and does not allow controlling for the effect of (partial and total) feature repetition and consequent priming phenomena. Indeed, as outlined above, to provide unequivocal evidence of real congruency and sequential effects, priming-free paradigms with a complete alternation sequence are required at least in first-order trials. However, as noted in Section "Confounding factors", it is impossible to have complete repetition-free sequences by using fewer than four possible responses. Indeed, with three responses, if there are two incongruent trials in a row, one feature must inevitably be repeated in the second trial. The influence of repetition effects appears to be even stronger in the spatial Stroop paradigms we discussed previously, since most of them used two possible alternative responses, in which only congruent-congruent sequences can be repetition-free. To solve this issue, Puccioni and Vallesi (2012a, b, c) designed a four-alternative forced choice spatial Stroop task, which has been shown to properly separate interference resolution from priming effects at least at first-order sequences (priming effects could in principle still be carried out in part from trials earlier than trial *n*-1).

In the last section of this review, we show that it is possible to design a spatial Stroop task that overcomes the outlined methodological limitations and provide some examples.

## Examples of complete spatial Stroop tasks

**Fig. 6:**
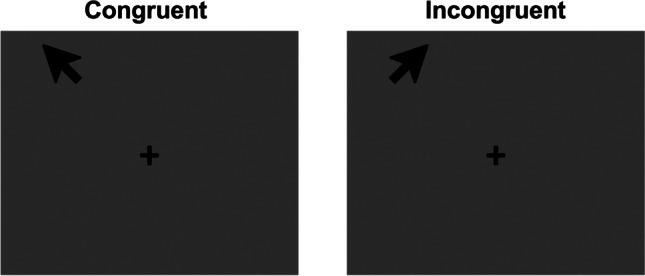
Example stimuli of the arrow-position spatial Stroop task designed by Puccioni and Vallesi (2012a). In the congruent condition (*left*), the arrow direction and position are both upper-left, while in the incongruent condition (*right*), the arrow direction is upper-right (task-relevant information) but appears in the upper-left position (task-irrelevant)

Puccioni and Vallesi (2012a) designed a spatial Stroop paradigm that satisfies the methodological requirements and overcomes the previously discussed limitations (see Fig. 6). The task was an arrow-position task consisting of an arrow pointing to four possible directions (upper right, upper left, lower right or lower left) that could appear in one of four positions on the screen (upper right, upper left, lower right or lower left). Participants were instructed to respond to the pointing direction of the arrow by pressing the corresponding key and ignoring the arrow position. Besides being purely spatial, this paradigm ensures a complete Stroop effect, as it encompasses all the required processing loci. Indeed, there is conflict between two different tasks, position and direction identification, with the former being stronger than the latter. Moreover, at the stimulus locus, there is a dimensional overlap between the relevant and irrelevant stimulus dimensions, since the arrows could appear in one of the four corners of the screen and point to one of the same four directions. Lastly, since the spatial arrangement of the response keys is compatible with the direction and position of the stimuli, the dimensional overlap between both stimulus dimensions and the response dimension was also ensured, implying that conflict/facilitation at the response level is complete. Furthermore, the presence of four arrows and four positions allows for a complete alternation of the stimulus feature across first order trial sequences, so that the direction and position of the stimuli in trial *n* always differ from the direction and position in trial *n*-1 (Puccioni & Vallesi, 2012b). Notably, in a previous study, we have found that its spatial Stroop effect has a good split-half reliability (0.767) (Capizzi et al., 2017) and that this task is suitable for being implemented with EEG (Ambrosini & Vallesi, 2017; Tafuro et al., 2019), also with mouse responses (Tafuro et al., 2020).

Puccioni and Vallesi's (2012a) paradigm is well suited to variations; indeed, alternative versions of it can be designed that allow satisfying the methodological criteria that we consider fundamental to have a complete Stroop effect. This is what we did in our recent study (Viviani et al., 2023), in which five new alternative spatial Stroop versions were evaluated, considering both the size and internal reliability of their Stroop effects. Specifically, the study aimed at finding an alternative spatial Stroop variant that is more suitable for neuroimaging studies. Indeed, although Puccioni and Vallesi's (2012a) paradigm fulfills all the methodological requirements for yielding a complete spatial Stroop effect, its peripheral spatial arrangement might be problematic during neuroimaging and electrophysiological (e.g., EEG) recordings, as it induces visuospatial attention shifts and a large extent of ocular artifacts.

A detailed description of the tasks is provided by Viviani et al. (2023), while in the present work we just want to highlight that methodologically complete spatial Stroop tasks are feasible. Indeed, all the new versions implied a three-level effect. First, all tasks were designed so that the processing of one dimension was stronger than the processing of the other to ensure a strong task conflict. Regarding this, we need to point out that processing asymmetry was obtained by leveraging the higher processing automaticity of one dimension relative to the other and/or the higher discriminability/perceptual salience of one dimension as compared to the other (see below for further details). The other two processing loci were also guaranteed to be involved because of the presence of all necessary dimensional overlaps. More in detail, in each of the new versions, the task-relevant feature was the direction of a target arrow, pointing to the upper-left, upper-right, lower-right, or lower-left part of the screen, as in Puccioni and Vallesi (2012a), and participants had to indicate this feature using four keys that were spatially arranged to ensure the dimensional overlap between the stimulus and response dimensions.

**Fig. 7:**
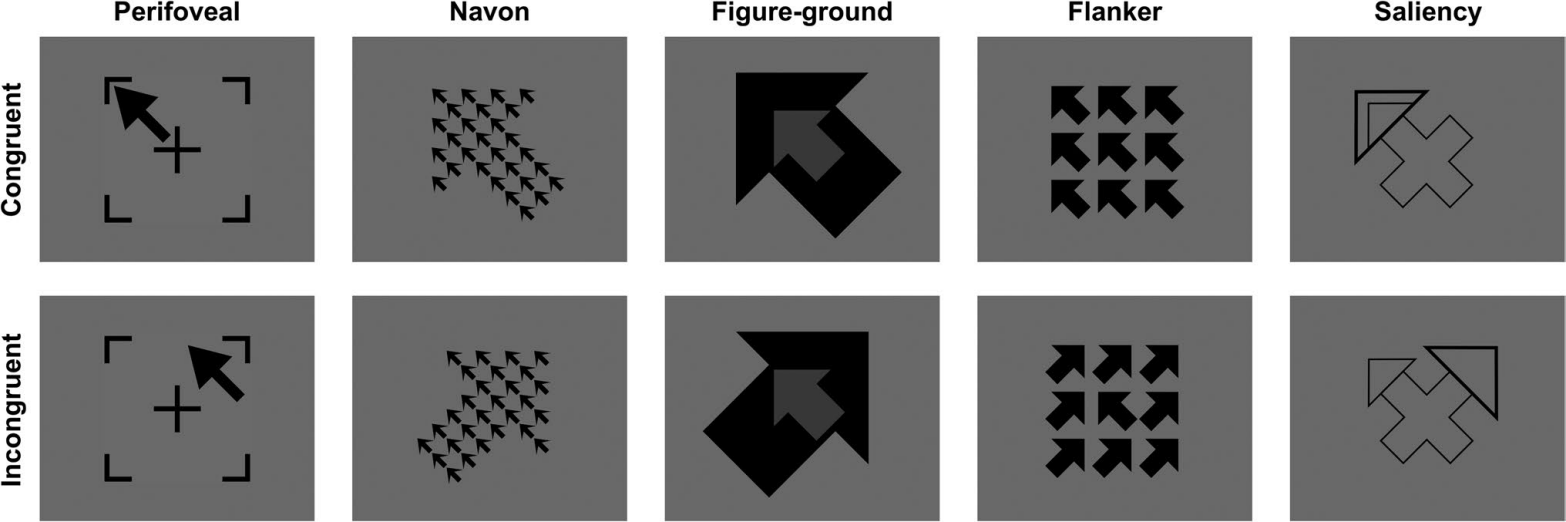
Example of the spatial versions of Stroop tasks proposed by Viviani et al. (2023). In congruent trials, the arrow direction and its position (in the Perifoveal) and the task-relevant arrow direction and the task-irrelevant arrow direction (in the other tasks) are both upper-left, while in incongruent trials, the arrow direction (task-relevant) is upper-left but the arrow position/direction (task-irrelevant) is upper-right

In the *Perifoveal Stroop*, the task-irrelevant information was the position generated by presenting the arrow in one of four small squares around the fixation cross. In the *Navon Stroop* version, task-relevant small arrows were spatially arranged to form a global arrow, whose direction was the task-irrelevant information, whereas in the *Figure-Ground Stroop*, the task-relevant small gray arrow was embedded in a large task-irrelevant black arrow. The *Flanker Stroop*^9^ consisted of a central arrow (task-relevant), flanked by eight arrows of the same size, which were task-irrelevant. Lastly, in the *Saliency Stroop* task, two empty triangles were added to an empty diagonal cross, the smaller indicated the task-relevant direction, whereas the bigger the task-irrelevant one. The reader is referred to Fig. 7 for examples of congruent and incongruent trials in each of the described tasks.

Therefore, there was always an overlap (perceptual or conceptual) between the relevant and irrelevant stimulus dimensions which, in the case of the Perifoveal Stroop, was between the arrow direction and its position whereas, in the remaining task versions, it was between the task-relevant arrow direction and the task-irrelevant arrow direction. Moreover, in all these variants, the stimulus attributes, both the relevant and irrelevant ones, overlapped with the response as the response keys were selected in order to be spatially compatible with the four directions and also with the positions in the case of the *Perifoveal* task. Of note, the *Peripheral* and the *Perifoveal Stroop* are arrow-position spatial Stroop tasks, whereas the other experimental paradigms do not belong to this category, and they could be better referred to as arrow-arrow spatial Stroop tasks, as the irrelevant dimension is the direction of the task-irrelevant arrow. It might be argued that in these four versions there are not two conflicting task sets, because both task-relevant and task-irrelevant dimensions imply arrow direction processing. However, task conflict is still present, with the only difference that, as claimed above, it was generated not only by leveraging processing automaticity but also by enhancing the perceptual discriminability of the task-irrelevant dimension as compared to the task-relevant one. Hence, in the arrow-arrow variants, two competing task-sets were still necessarily activated by the conceptually or perceptually different characteristics of the two dimensions. For example, in the Navon Stroop, task-related conflict relies on a mix of conceptual and perceptual characteristics, as there is a stronger but task-irrelevant processing stream elaborating the global arrow direction which competes with the less strong but task-relevant processing of local arrow directions. Hence, global vs. local processing is a perceptual characteristic which, however, has also conceptual implications. In contrast, in the Saliency Stroop task, task conflict is driven solely, as the name suggests, by different degrees of saliency between the task-relevant (less salient) and task-irrelevant (more salient) dimensions. This difference notwithstanding (see Viviani et al., 2023, for a more detailed discussion), the Peripheral and all the novel versions are pure spatial Stroop variants including all the conflict levels.

Lastly, the new versions also satisfied the second methodological point, as they all entailed four relevant and four irrelevant stimulus dimensions, allowing to completely alternate the first-order trial sequences, thus reducing low-level binding and priming effects^10^.

## Summary and Conclusion

This study emerged from the need to emphasize the importance of measurement validity in assessing the Stroop effect. While the validity of measurements is undeniably crucial in psychology, it is consistently threatened in the context of the Stroop task literature, mainly due to significant methodological differences across studies. This, in turn, has led to theoretical controversies. The methodological variability comes from the existence of an incredible number of Stroop task variants, often created without adhering to shared guidelines. Therefore, the aim of the present work was to highlight the importance of using rigorous methodological criteria to design Stroop tasks that measure the Stroop effect in a comprehensive and valid way.

In this review, we started with an overview of the classical Stroop effect, highlighting its complex nature, and presenting evidence that demonstrates that it is composed of effects arising at multiple processing levels or loci. Therefore, throughout this work, we stressed that designs generating conflict at the task locus and interference/facilitation at the stimulus and response loci are fundamental to provide complete measures of the Stroop effect, that is, measures that consider such an effect as a whole. We also showed that, in order to meet these requirements to be satisfied, a Stroop task should adhere to the specific characteristics elegantly summarized in Kornblum’s works which, although rarely used, provide highly useful practical guidelines in the design of Stroop tasks. Furthermore, we highlighted the role of possible confounding factors, such as repetition effects, which should be controlled for (e.g., by using priming-free designs).

After discussing the most popular alternative versions of the Stroop task, we concluded that most of them did not entail the possibility to yield complete Stroop effects. As a result, they cannot be defined as Stroop tasks, as they differ from the classical color–word Stroop task. Indeed, we believe that to ensure validity, each replication of this task should aim to maintain methodological consistency with the classical Stroop task. Only this ensures that meaningful comparisons can be made between evidence produced by different studies.

However, while emphasizing the importance of methodological aspects and the validity of Stroop effect measures in future studies, we did not intend to imply that only classical Stroop tasks should be employed. Instead, we proposed an alternative category of Stroop tasks, namely the spatial variant, as an example of an alternative Stroop version that maintains methodological adequacy, while also offering increased flexibility in specific cases. We thus provided a methodological review of the spatial Stroop tasks in the literature to verify whether they satisfy such criteria. However, we also found that the majority of the spatial Stroop paradigms implemented in the literature lacked response-related effects and, thus, did not ensure a complete Stroop effect.

First, the label spatial Stroop was also referred to non-purely spatial tasks. This is an issue since including verbal stimuli does not allow either to fully leverage the advantages related to the use of exclusively spatial stimuli or to have the required dimensional overlaps to produce response-related effects. Therefore, we suggested that only arrow-position tasks were ideal spatial Stroop paradigms, in the sense that they allowed one to totally exclude linguistic processing. A second fundamental problem was that even among the arrow-position spatial Stroop tasks in the literature, some of them did not ensure a complete spatial Stroop effect, mainly due to the absence of stimulus–response overlaps. Thus, the majority of the discussed paradigms were not classifiable as complete Stroop tasks but fitted better the more cautious definition of Stroop-like paradigms (Kornblum, 1992). A third issue was more general and concerned all the Stroop tasks, that is, the need of using at least four stimulus dimensions in order to control for first-order low-level binding and priming effects.

On the basis of these methodological considerations, we provided some examples of spatial Stroop tasks, which allow one to yield complete spatial Stroop effects and to exclude the influence of trial sequence confounding effects. Nevertheless, this work wants to stress that these paradigms are not the only possible spatial Stroop variations and that, by satisfying the above methodological considerations, several different variations can be conceived and designed. For this reason, our categorization of spatial Stroop tasks is not exhaustive, and arrow-position tasks are not the only purely spatial Stroop paradigms. For example, we showed that other pure variants can be created, such as some of those presented in Viviani et al. (2023), which were not arrow-position tasks, but still satisfied the main methodological criteria and yielded large and reliable Stroop effects. Our results indicate that, when using tasks that are methodologically comparable to the classical Stroop task, not only the measure validity but also its reliability was ensured, showing that the Stroop effect can be large and reliable at the same time, in contrast with the issue posed by the reliability paradox.

Overall, although the current literature on spatial Stroop tasks has some methodological limitations, the spatial Stroop represents a valid and promising alternative to the color–word Stroop task and to its several variations. However, careful attention must also be paid when designing spatial Stroop experimental paradigms to satisfy the methodological criteria whose importance was stressed in the present work. In summary, spatial Stroop tasks should (i) be purely spatial and avoid verbal stimuli, (ii) ensure conflict at the level of task, as well as conflict/facilitation at the stimulus and response loci, and (iii) control for repetition effects, at least at first-order trial level, thus using four (or more) stimuli and responses.

However, we want to emphasize that, in proposing the spatial Stroop task as a valid Stroop task variant, we do not intend to imply that it is the only potentially valid alternative. While this review has specifically focused on spatial Stroop due to its ability to exclude certain potentially confounding factors (e.g., the use of linguistic stimuli) and its reliance on universally recognized automatic tasks (e.g., identifying position), other variants may also meet the required methodological criteria. Furthermore, by providing examples of spatial Stroop tasks, our intention was to demonstrate the underlying rationale in a practical manner, with the aim of encouraging other scholars to do the same, while also using different Stroop paradigms.

To conclude, fulfilling these methodological criteria is important because they represent the only means to obtain truly comparable measures of the Stroop effect. As a consequence, if more rigorous task designs are employed, there will be more room for enhancement in the understanding of processes tapped by the Stroop task. Indeed, starting from the same design criteria would ensure that the Stroop effect measures of different studies actually reflect the same phenomenon, and not only a part of it (e.g., the effects at stimulus level), and not confounded by priming effects due to (partial and total) feature repetitions. The take-home message of the present work is in line with other recent works (e.g., Augustinova et al., 2019; Parris et al., 2022), that have highlighted that the nature of the Stroop effect is much more complex than previously expected. Therefore, since there is evidence that the Stroop effect occurs at multiple loci, there is a clear need of designing experimental paradigms capturing all the different types of underlying processes and not just a part of them. To attain a more thorough and comprehensive comprehension of the extensively studied Stroop effect, it is imperative to implement more rigorous methodological practices within the (spatial) Stroop literature. Enhancing measurement validity stands as the sole pathway to achieve this goal.

## Data Availability

Not applicable.

## Footnotes

1 Our intention is not to conduct a systematic review of the literature; hence, we explicitly declare that we avoid doing a detailed and comprehensive search on the literature on the Stroop tasks, which is the requisite of any systematic review (e.g., Uman, 2011). With this, we mean that we do not follow the PRISMA statement (Page et al., 2021), necessary for any systematic review.

2 We will use the terms conflict and interference as synonyms for the sake of simplicity.

3 To note that, as compared to previous works, in Parris et al. (2022), the possible contribution of multiple processing loci has been investigated considering separately Stoop interference and Stroop facilitation. In brief, both imply interference at the task locus, whereas SI also relies on interference at the stimulus and response loci and SF also relies on facilitation at the stimulus and response loci. Therefore, we could say that both SI and SF imply the same processing loci, but with an opposite direction at the stimulus and response loci. Based on that, given our intention to focus on the overall Stroop effect, in the present work, when possible, we will avoid referring to interference and facilitation at these loci. We will instead use more caution, referring in a general manner to the processing loci of the Stroop, except for task conflict which can be safely used.

4 It is here important to note that a complete alternation sequence at first-order trials (*n*-1) allows only to reduce binding and priming effects. Indeed, to completely exclude such effects, the repetition sequence of trials earlier than trial *n*-1 should be taken into account to completely exclude possible carryover effects.

5 Other sub-variants of this task can also be used, such as those asking for categorization, that is, tasks requiring participants to identify the semantic category of the picture instead of naming it (e.g., Mayor et al., 1988). Picture–word Stroop versions demanding for tasks other than naming are not discussed here because in our view they differ by nature from the classical Stroop tasks since they, for example, necessarily imply lower degrees of overlaps (e.g., considering the entire category and not a single item reduces the overlap between the relevant and irrelevant stimulus dimensions).

6 In fact, it also depends on the size of the stimulus set, which is necessarily limited for the color–word Stroop task, whereas can be potentially unlimited for the picture–word Stroop task.

7 Nevertheless, in the literature, this distinction is not that clear. Indeed, some tasks can be placed in-between these two distinct forms. For example, Spinelli et al. (2019 ) used a paradigm that here we defined as picture–word Stroop task but, like in picture–word interference tasks, they used more than one semantic category. Therefore, in congruent trials they had proper Stroop stimuli (the word and the picture both refer to the same concept), while in incongruent ones, in which the word was superimposed onto semantically unrelated pictures, the semantic relevance was necessarily lower.

8 According to Kornblum’s taxonomy, for spatial Stroop tasks employing only spatial stimuli to obtain a type-eight ensemble, responses must be manual (to ensure the overlap between the irrelevant dimension and the response). Therefore, the spatial Stroop effect inevitably includes the Simon effect (representing a type-three ensemble, thus lower in the hierarchy of overlaps), but this does not mean that the Stroop effect is made less pure by the effect of lateralized responses. Rather, lateralized responses are fundamental to ensure the required stimulus–response overlaps and, when responses to spatial stimuli are made vocally, only type-four ensembles and Stroop-like tasks are obtained. It is also important to note that type-eight-ensemble spatial Stroop tasks also inevitably include the flanker effect (representing a type-four ensemble), which derives from the required stimulus–stimulus overlap.

9 It might be argued that the flanker Stroop cannot be considered a Stroop adaptation, since the task-relevant stimulus is not spatially overlapped with the task-irrelevant ones. However, although there is evidence that interference is reduced when task-relevant and task-irrelevant features are spatially separated (Lamers & Roelofs, 2007), our flanker Stroop task can still be considered a Stroop task. Indeed, according to Kornblum’s taxonomy, the overlap is not necessarily perceptual, but it can be conceptual as well. Therefore, as long as the task-relevant and task-irrelevant stimuli can be perceived as an ensemble (as in our case, in which they are minimally distant and perceivable in perifoveal vision), the conceptual overlap is guaranteed. As such our flanker Stroop is a type-eight ensemble thanks to the presence of task conflict (generated between the processing of the numerous flanking stimuli and the processing of a single feature, see also Viviani et al., 2023), stimulus conflict (due to stimulus–stimulus overlap) and response conflict (due to stimulus–response overlap generated by the compatible spatial arrangement of response keys). Of note, we named it flanker Stroop based on the presence of flanking task-irrelevant stimuli, but we highlight its fundamental difference from classic flanker tasks (which are type-four ensembles), that is those formally known as Eriksen flanker tasks (Eriksen & Eriksen, 1974).

10 Four stimulus–response mappings are sufficient to reduce first-order trial repetitions, as outlined above, but it does not have to be intended as the only possible alternative. Indeed, to minimize repetition effects, one needs at least four stimuli and responses, but this represents only a lower bound, since more than four options could also be employed. Indeed, specifically considering the spatial Stroop task, the maximum possible number of stimulus–response overlaps coincides with the maximum number of possible effectors (e.g., the fingers). For example, using more than four stimulus–response mappings could be preferable for controlling for those repetition carryover effects that can be potentially present even if first-order trials are repetition-free. Indeed, as outlined above, to totally control for repetition effects, one should also avoid repetition in the trials earlier than trial *n*-1, and to do so, the number of stimulus–response mappings has to be increased (of course at the prize of increasing Stimulus–response mapping complexity). Overall, priming-free Stroop tasks are desirable, at least at first-order trial level, and this can be achieved by using at least four options, while using more than four mappings is a researcher’s choice and depends on the experimental question, on the need of controlling for longer carryover effects, and on the feasibility of using complex Stimulus–response mappings in different experimental contexts (including the type of research participants, e.g., healthy younger adults, neurological patients, older adults, etc.).
